# The impact of the parenting for respectability programme on violent parenting and intimate partner relationships in Uganda: A pre-post study

**DOI:** 10.1371/journal.pone.0299927

**Published:** 2024-05-24

**Authors:** Godfrey Siu, Rebecca N. Nsubuga, Jamie M. Lachman, Carol Namutebi, Richard Sekiwunga, Flavia Zalwango, Julie Riddell, Daniel Wight

**Affiliations:** 1 Child Health and Development Centre, School of Medicine Makerere University College of Health Sciences, Kampala, Uganda; 2 MRC/UVRI & LSHTM Uganda Research Unit, Entebbe, Uganda; 3 Department of Global Health, LSHTM, London, United Kingdom; 4 MRC/CSO Social and Public Health Sciences Unit, University of Glasgow, Glasgow, Scotland; 5 Department of Social Policy and Intervention, University of Oxford, Oxford, United Kingdom; 6 University of Cape Town, Cape Town, South Africa; Taipei Veterans General Hospital, TAIWAN

## Abstract

**Background:**

There is a growing need for interventions that reduce both violence against children and intimate partner violence in low- and middle-income countries. However, few parenting interventions deliberately address this link. We tested the feasibility of a 16-session group-based parenting programme, *Parenting for Respectability*, in semi-rural Ugandan communities.

**Methods:**

This was a pre-post study with parents and their children (*N* = 484 parents; 212 children).

**Results:**

Pre-post comparisons found large effects for parent-reported reduced harsh parenting (Cohen’s *f*^2^ = 0.41 overall; *f*^2^ = 0.47 (among session attendees); with an overall reduction of 26% for harsh parenting. Session attendees reported higher reductions than non-attendees (p = 0.014), and male caregivers reported higher reductions than female caregivers (p<0.001). Children also reported reduced harsh parenting by attending fathers (*f*^2^ = 0.64 overall; *f*^2^ = 0.60) and attending mothers (*f*^2^ = 0.56 overall; *f*^2^ = 0.51); with reduction in harsh parenting ranging between 27% to 29% in the various categories. Overall, spousal violence reduced by 27% (*f*^2^ = 0.19 overall; *f*^2^ = 0.26 (among session attendees). Both parents and children reported reduced dysfunctional parent relationships; parents: *f*^2^ = 0.19 overall; *f*^2^ = 0.26 (among session attendees); and children: *f*^2^ = 0.35 overall; *f*^2^ = 0.32 (for attending parents); with reductions ranging between 22% to 28%. Parents who attended more than 50% of the program reported greater effects on reduced dysfunctional relationships than those who attended less than half of the program (*B* = -0.74, p = 0.013). All secondary outcomes were improved with *f*^2^ ranging between 0.08 and 0.39; and improvements ranging between 6% and 28%.

**Conclusion:**

Results suggest the importance of more rigorous testing to determine program effectiveness.

## Introduction

Intimate partner violence (IPV) and violence against children (VAC) are interlinked and are major social, development and public health concerns. Globally it is estimated that approximately 30% ever-partnered women worldwide have experienced physical and/or sexual violence by an intimate partner at some point in their lives [1]. IPV prevalence among women in Uganda is very high. The Uganda Demographic and Health Survey 2018 found that 36% of women had ever experienced partner physical violence, while 22% had ever experienced partner sexual violence [2]. Violence against children is extremely widespread globally, with approximately half of all children–one billion aged 2–17 years–reporting having experienced violence in the past year [3]. The Uganda national VAC survey 2015 found that 59% girls and 68% boys had experienced physical violence in their childhood, and 35% girls and 17% boys had experienced sexual violence in their childhood [4]. Such violence in Uganda and most Sub-Saharan African countries is usually perpetrated by people known to children in their homes and community [5,6]. IPV and VAC are major causes of morbidity and mortality, they undermine the social functioning of the victims and their families, and have lifetime consequences for physical, sexual, reproductive and mental health [7,8]. The prevention of both forms of violence would contribute to many Sustainable Development Goals since they strain health systems, lower educational achievement and economic productivity, and undermine economic and social development, [9–11] and elimination of IPV is essential to Goal Five.

Many studies confirm the link between VAC and IPV, suggesting the need for an integrated approach to their prevention. A review by Geudes and colleagues identified six ways in which they are interrelated: they have many shared risk factors, starting in the family; social norms legitimise both and discourage children and women from seeking help; they often occur within the same household; both can be transmitted across generations; they can have similar consequences across the lifespan, and finally, both intersect in adolescence, a time of heightened vulnerability to violence [12].

Factors perpetuating IPV and VAC exist at multiple socio-ecological levels. For IPV, familial level factors include having been abused as a child, having an absent or rejecting father, inter-partner conflict, and male control of wealth and decision-making. Community level factors include women’s isolation and male peer groups that legitimize men’s violence. At the macro level IPV is associated with cultural norms that condone violence within the family, schools and community, establish rigid gender roles and link masculinity to toughness, male honour, dominance and ownership of women, and it thrives where policy, legislation and implementation of laws is weak [13].

The shared familial risk factors for IPV and VAC, and their origin in adverse early exposure, provide a great opportunity for early intervention through optimizing parental influence. An increasing number of parenting programs are therefore being implemented and tested in Low and Middle Income Countries (LMICs) to reduce VAC [14], and substantial evidence currently shows that if delivered with fidelity and systematically, they can be effective in improving child outcomes [15,16]. However, interventions directly addressing early prevention of both IPV and VAC in LMICS remain limited [17].

Furthermore, very few parenting programs in LMICs harness cultural drivers and pre-existing motivations to change behavior. In sub-Saharan Africa little attention has been paid to one of the most important dimensions of parenthood for both mothers and fathers: the need to maintain the family’s respectability, in large part achieved through the appropriate behavior of the children and their parents [18,19]. This core motivation might be harnessed in the design of interventions to reduce spousal violence, modify negative parenting and encourage sensitive parenting, in order to reduce children’s future risk of sexual, physical and/or emotional violence. In Uganda, we are not aware of parenting programs that deliberately recruit parental couples to complete both single and mixed sex sessions. We therefore designed a community-based parenting program,–*Parenting for Respectability* (*PfR*),–to address this gap in Uganda, and contribute evidence on how a parenting program can address both IPV and VAC. Following careful formative evaluation [20] we conducted a pre-post study to establish whether there was sufficient evidence of effectiveness to warrant progression to a randomized controlled trial. This paper reports on this study.

### Study objective

The objective was to generate initial quantitative evidence of the effectiveness of the *Parenting for Respectability* Program in modifying key outcome measures on caregiving, parent-child relationships and relationships between partners and intimate partner violence (IPV). Through this project we tested the program to provide further evidence of program acceptability, the plausibility of the measures, the intended mechanism of change and the effects of the intervention on key outcomes associated with sexual and gender-based violence, in particular violence against children. The project was delivered in central Uganda, near Kampala city, in collaboration with SOS Children’s Village, a local NGO working in Wakiso District.

## Methods

### Study design

We conducted a pre-post study in fourteen villages from three sub-counties in Wakiso District, Uganda. Kakiri and Ssisa sub-counties are basically rural, with subsistence farming the main economic activity, while Katabi is peri-urban with informal trade as the main activity. Six hundred and seventy (670) parents were approached during mobilization, out of whom 645 parents were interviewed using a questionnaire at baseline, and registered as potential participants in the group-based intervention. Baseline data were collected in 2017 from consenting parents and half of the number of parent participants provided a list of their children aged 8–15 years old from whom one child was selected randomly for interviews, providing parent-child data. The *PfR* sessions were then delivered to the parents for four months, and follow-up data collected three months post intervention completion in 2018.

### Participants

Parents were first informed about the PfR Programme through local leaders, who went door-to-door, with support from the project team. Those interested in learning more were invited to a group meeting at the community centre, to receive study information. Anybody who identified as a parent, either biological parents or caregivers, was eligible as long as they were currently parenting children aged 0–17 years. The programme particularly emphasized the recruitment of fathers and parental couples. Once groups were recruited, the programme was then delivered once a week for 15 weeks by local facilitators selected from amongst the parents.

### Data collection, management, and ethical procedures

Trained and supervised research assistants conducted informed consent procedures and collected demographic and outcome data using paper-based questionnaires administered orally in local languages to account for low-literacy rates. Data was entered into an OpenClinica software database with data cleaning conducted using Stata 15. The study protocol was approved by the ethics review boards of Uganda Virus Research Institute (GC/127/18/02/584) and Uganda National Council for Science and Technology (SS 4228) and at the University of Glasgow.

### The parenting for respectability program

The Parenting for Respectability Programme (PfR) was developed over five years following the Six Steps for Quality Intervention Development (6SQuID) model [21]. It initially underwent formative evaluation over two years (2014–2016) with six groups in three villages in Wakiso District. *PfR* is a 16-session manualized program starting with nine single sex sessions followed by seven mixed sex sessions, delivered once a week by two local facilitators who receive one week’s training. The program draws on parents’ pre-existing motivation to maintain respectability, largely achieved through children’s good behavior and respect for elders, and builds on parents’ existing skills and experience. The program addresses four familial processes associated with poor parenting and intimate partner violence: poor parental bonding and child attachment; harsh parenting; inequitable socialization by gender and parental conflict. A particular goal is to involve fathers, whom most parenting programs find hard to recruit, and the first nine sessions are delivered in single-sex groups, as a way to encourage more fathers to attend. The intervention’s rationale, program theory and formative evaluation are described elsewhere [22].

### Measures

#### Demographic outcomes

Demographic data for parents and children were collected at baseline. Family characteristics included parent/child sex and age; caregiver relationship to child; type of caregivers in each household; child school attendance; and household size, type of household, income, and hunger (e.g., frequency in past month that family experienced went to bed hungry).

#### Primary outcomes

Primary outcomes for this study were changes in level of **harsh parenting** (parent- and child-report) and **dysfunctional partner relationships**, in particular, spousal violence (parent- and child-report). Parent-report (6 items) and child-report (8 items) of **harsh parenting** was based on the frequency of verbal and physical abuse in the past month (e.g., “How often do you hit your child with a stick or other objects when he/she has done something wrong?”) with items ranging from 1 (never) to 4 (often). Children reported on both their female and male caregivers which were analysed separately. Parent-report (9 items) and child-report (4 items) of **dysfunctional partner relationships** was based on the frequency of verbal and physical conflict (spousal violence) between male and female partners (e.g., “How often is there serious anger or hostility between you and your partner”) with each item ranging from 1 (not at all) to 4 (more than once a week).

#### Secondary outcomes

Secondary outcomes included positive parenting (parent-report: 18 items; child-report: 15-items), respectful child behavior (parent/child-report: 4 items), career-child conflict (parent-report: 5 items), parent self-inefficacy (parent-report: 4 items), provision of necessities as proxy for child neglect (parent/child-report: 6/14 items), co-parenting (parent-report: 4 items), partner involvement in caregiving (parent-report: 5 items), and community/collective parenting (parent-report: 3 items), attitudes towards gender socialization (parent-report: 13 items), and knowledge of child development (parent-report: 5 items).

All measurements were previously validated in Wakiso during a formative evaluation study in 2018, with psychometric properties being examined further using baseline assessments. Table 1 summarizes the outcomes assessed at baseline and post-test.

**Table 1:** Summary of outcomes assessed at baseline and post-test.

Outcome	Source	No. of Items	Reliability^1^
Primary outcomes			
Harsh parenting	Parent	6	0.61
Harsh parenting–male carer	Child	8	0.72
Harsh parenting–female carer	Child	8	0.62
Dysfunctional partner relationships	Parent	9	0.78
Dysfunctional partner relationships	Child	4	0.85
Secondary outcomes			
Positive parenting	Parent	18	0.84
Positive parenting–male carer	Child	14	0.91
Positive parenting–female carer	Child	15	0.84
Parent sense of inefficacy	Parent	4	0.45
Caregiver-child conflict	Parent	5	0.57
Respectful child behavior	Parent	4	0.6
Respectful child behavior	Child	3	0.68
Provision of child necessities	Parent	6	0.6
Provision of child necessities–male carer	Child	4	0.82
Provision of child necessities–female carer	Child	4	0.67
Co-parenting arrangements	Parent	4	0.53
Partner involvement	Parent	5	0.71
Community parenting	Parent	3	0.56
Gender socialization–attitudes	Parent	13	0.67
Knowledge of child development	Parent	5	0.3

### Process outcomes

Participation data were collected by program facilitators using attendance registers. Enrolment referred to whether participants attended at least one group session out of the total number of sessions. Attendance rates were calculated based on enrollees only [23].

#### Sample size calculations

A total of 645 parents and 261 of their children participated in the baseline survey, following two rounds of participants’ recruitment. We initially recruited 400 parents and 182 children for the study but out of this, 161 parents dropped out from the program after minimal involvement due, largely, to the unmet expectations for material benefits despite this having been explained at recruitment. To maintain a sufficient study sample, we recruited a further 245 parents and 79 children. With 645 parents, assuming a 5% level of significance and standard deviation of the change in an outcome of as high as 1 we would be able to detect an effect of *PfR* as low as 0.11 with 80% power. We used the sample size formula of Rosner for before-and-after studies based on a paired T-test [24].

#### Data analysis

Frequencies for several socio-demographic and risk factors were calculated to assess their distribution. We conducted confirmatory factor analyses (CFA) to assess if the items within each construct held together. Each construct contained varied number of items ranging from 3 to 18 for both the primary and secondary outcomes. After the CFA we performed a reliability analysis using the Cronbach’s Alpha [25] method to ascertain if the items within a given construct conferred internal consistency. An alpha of at least 0.7 was indicative of good reliability. Once the items within in each construct confirmed internal consistency, we generated scores for each construct variables for both the primary and secondary outcomes calculated by summing the scores assigned by each respondent on all items per construct. The scores ranged between 0 (e.g. never) to 4 (e.g. always). We assessed if these scores were normally distributed because the methods we were planning to use for the follow-on analyses have the assumption of normality. We used exploratory analysis methods e.g. summary statistics (comparing the mean and median), histograms and symmetry plots to assess normality. The results confirmed that the scores passed the normality test.

Linear mixed models accounting for random effects on the village and individual level were used to examine changes from baseline to post-test on primary and secondary outcomes. Fixed factors included assessment time point, child/caregiver gender, and caregiver age, allowing for analyses of differential effects by gender and age as well. Full information maximum likelihood estimation method was used to account for missing data. The analysis involved only parents with both baseline and post-test data. To assess relevance of outcomes, tests for precision of effect sizes were undertaken (i.e., 95% confidence intervals did not overlap zero), as well as the direction and magnitude of bias corrected Cohen’s f^2^ effect sizes. An effect size of 0.02 was considered small, 0.15 moderate, and 0.35 or greater, large [26]. Finally, we examined the impact of program enrolment and attendance on primary outcomes by adding either a dichotomous variable for enrolment (yes/no) or for those who attended either more or less than 50% of the 15 sessions to the statistical models. The level of significance was set at 5%. All the models were validated to assess if they conferred goodness of fit to the data by computing fitted scores and comparing them with the scores for each construct. We also checked if the residuals were normally distributed. All analyses were run using the STATA software version 15 (27).

### Ethics statement

The study was reviewed by the Research and Ethics Committee of the Uganda Virus Research Institute (UVRI) and thereafter approved by the Uganda National Council for Science and Technology (UNCST). Adult participants provided written informed consent following the administration of the information sheet that described the study purpose, procedures, data management, and their rights to voluntarily participate. Those who were not able to sign the consent form provided a thumb print. Parents consented for their children, but before being interviewed, children were asked to assent and were assured of protection in case they did not wish to participate or decided to end their participation during the course of the interview.

## Results

### Study flow

#### Socio-demographic characteristics of the sample at baseline

**Fig 1:**
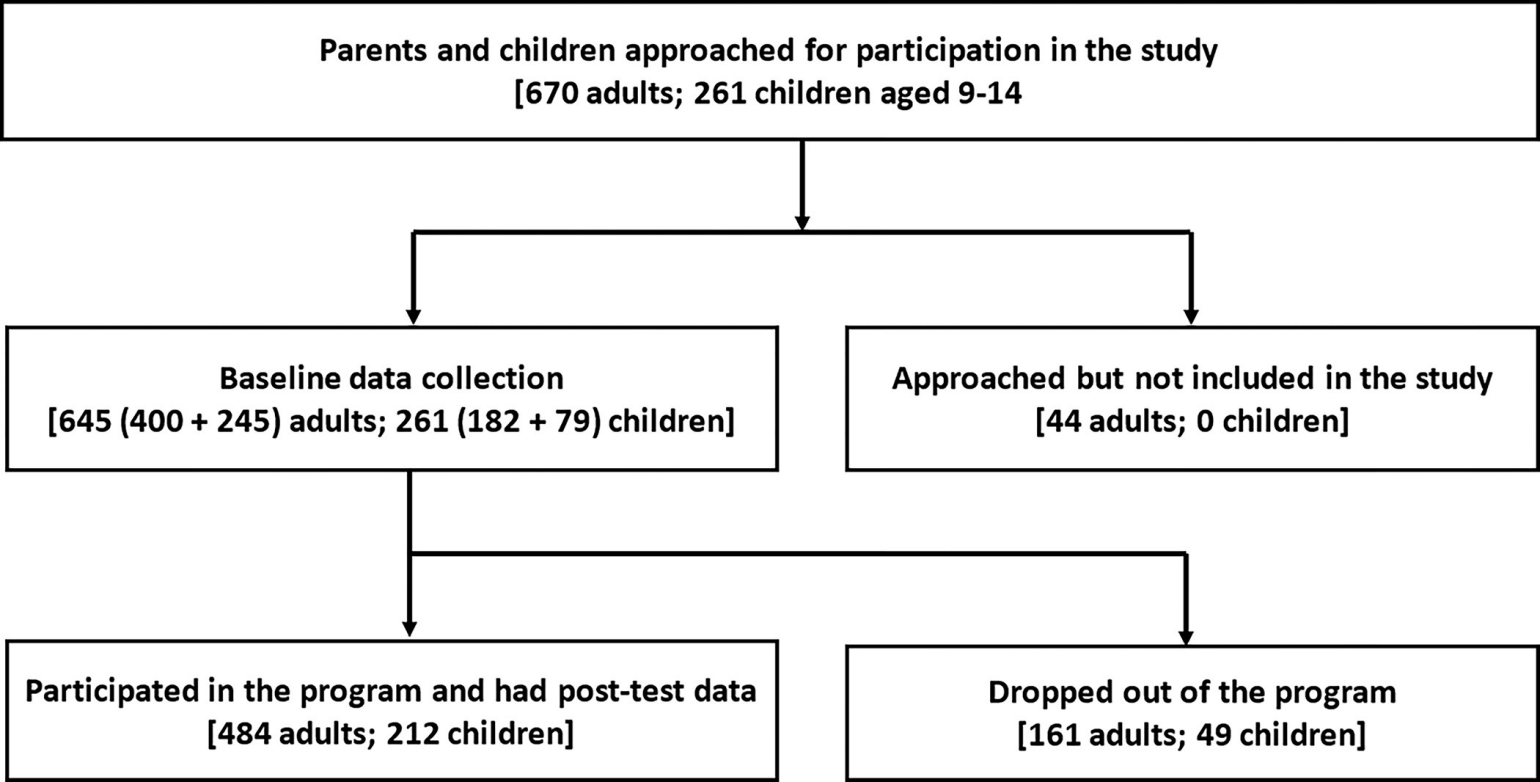
Participants’ flow diagram.

A total of 484 parents (*N* = 269 mothers, 215 fathers) and 212 children (*N* = 117 girls, 95 boys) completed the 3-months post-test survey as illustrated in participants’ flow diagram in Fig 1 above. Distributions of the socio-demographic and risk factors are presented in Table 2. Caregiver age ranged between 18 and 82 years with a mean of 39, SD (10.9). Children were between 8 and 15 years, with 65% below 12 years. Enrolment rate was 87% (421/484) parents attending at least 8 of the 15 *PfR* sessions; while 15 (3%) parents attended all the sessions. Out of the 212 children, 175 (82%) were linked to a household from the parents’ database; and 154 (88%) had a caregiver who attend at least one session.

**Table 2:** Characteristics of the sample at baseline.

Characteristic	Freq/Mean
**Parents (N = 484)**	
Sex, *n* (%)	
Female	269 (56.0)
Male	215 (44.0)
Age, M (SD)	38.9 (10.9)
Marital status, *n* (%)	408 (84.5)
Married	75 (15.5)
Single/Widowed/Separated	
Education level	
None/Incomplete Primary	205 (42.4)
Complete Primary and above	278 (56.6)
Employment, *n* (%)	
Farmer	249 (51.4)
Non-farmer	235 (48.6)
**Children (N = 212)**	** **
Sex, *n* (%)	
Female	117 (55.2)
Male	95 (44.8)
Age, M (SD)	11.7 (1.5)
Relationship to parent, *n* (%)	
Both parents	124 (66.3)
Single parent	47 (25.1)
Other/step/nonbiological	16 (8.6)
Enrolled in school, *n* (%)	201 (94.8)
**Household**	** **
Respondent is only caregiver in household, *n* (%)	62 (12.8)
Other parent lives in household (*N* = 422), *n* (%)	355 (84.1)
Number of children, M(SD)	
Girls	2.1 (1.7)
Boys	2.2 (1.6)
Total	4.3 (2.3)
Electricity in house, *n* (%)	234 (49.1)
Piped water in compound, *n* (%)	86 (17.8)

### Primary outcomes

**Fig 2:**
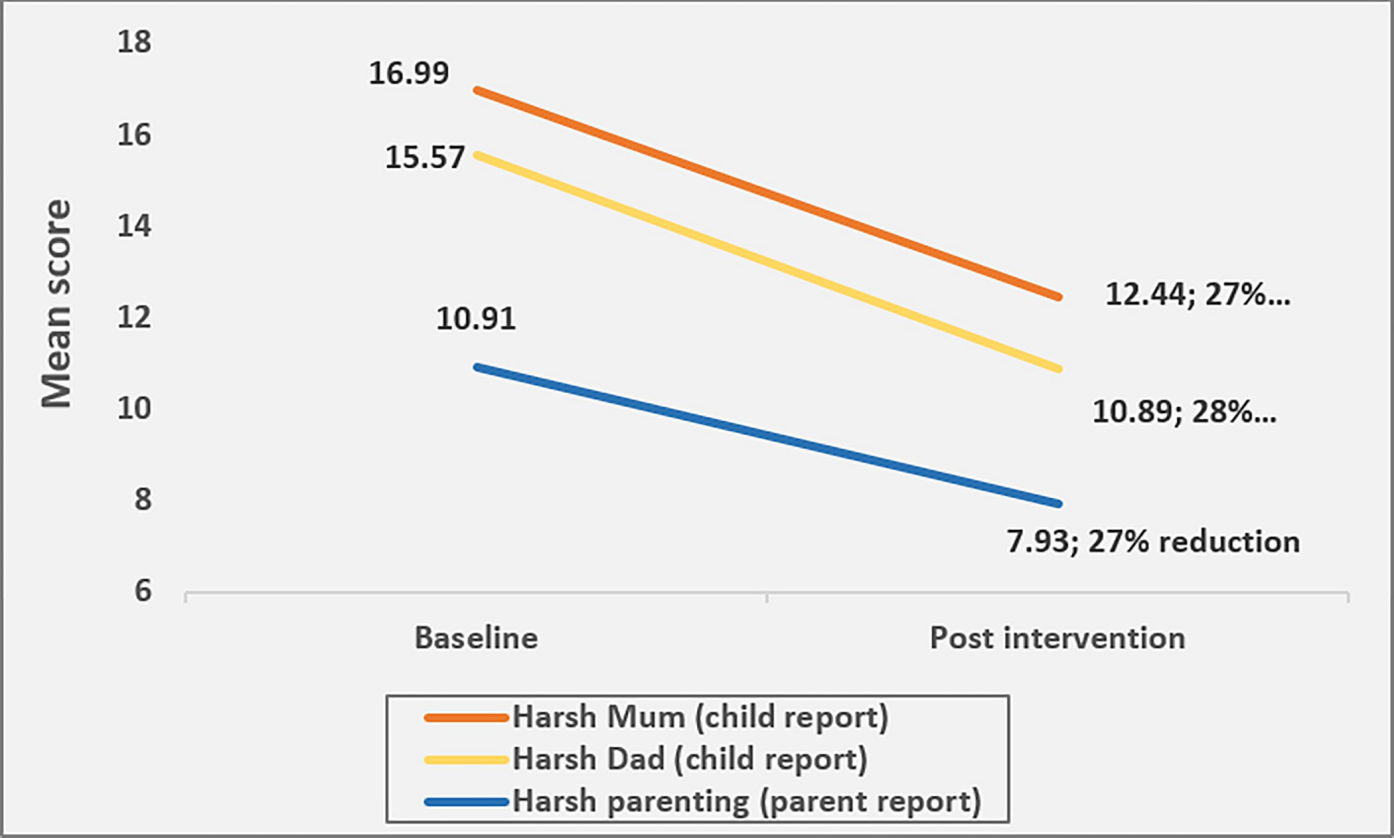
Changes (adjusted) from baseline to post-test for harsh parenting.

**Fig 3:**
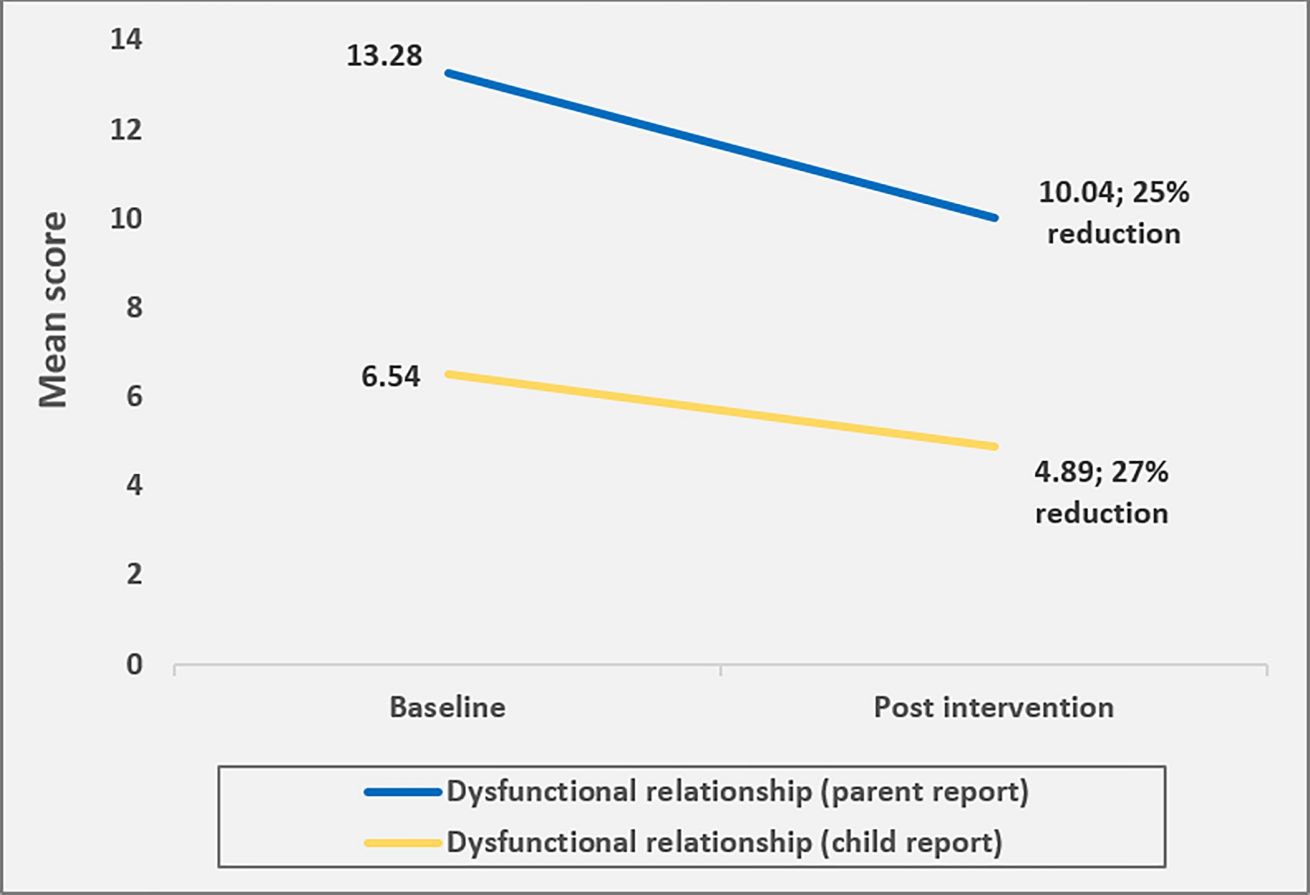
Changes (adjusted) from baseline to post-test for dysfunctional partner relationships.

Results for primary outcomes are summarized in Table 3 and Figs 2 and 3.

**Table 3:** Summary statistics, effect estimates, and effect sizes for primary outcomes.

				Baseline		Post-test					
Outcome	Report	Model	N	Range	Mean (SD)	Range	Mean (SD)	Effect (95% CI)	p-value*	% change	Cohen f^2^
Harsh Parenting	Parent	0 vs 1+ sessions	481	[1,20]	10.90 (2.96)	[5,17]	8.08 (2.18)	-2.83 (-3.13, -2.52)	<0.001	26%	0.41
		1–7 vs 8+ sessions	419	[1,20]	10.91 (2.98)	[5,17]	7.93 (2.09)	-2.97 (-3.28, -2.66)		27%	0.47
Harsh Mother	Child	0 vs 1+ sessions	171	[9,28]	17.17 (3.91)	[6,25]	12.53 (3.55)	-4.63 (-5.78, -3.47)	<0.001	27%	0.56
		1–7 vs 8+ sessions	151	[9,28]	16.99 (4.02)	[6,25]	12.44 (3.49)	-4.55 (-5.72, -3.38)		27%	0.52
Harsh Father	Child	0 vs 1+ sessions	142	[8,27]	15.77 (4.39)	[1,23]	11.17 (3.44)	-4.55 (-5.36, -3.73)	<0.001	29%	0.64
		1–7 vs 8+ sessions	124	[8,26]	15.57 (4.28)	[1,22]	10.89 (3.21)	-4.40 (-5.23, -3.57)		28%	0.6
Dysfunctional relationship	Parent	0 vs 1+ sessions	429	[4,26]	13.13 (3.68)	[0,27]	10.25 (3.25)	-2.89 (-3.31, -2.47)	<0.001	22%	0.19
		1–7 vs 8+ sessions	375	[4.26]	13.28 (3.75)	[0,27]	10.04 (2.89)	-3.27 (-3.69, -2.85)		25%	0.26
Dysfunctional relationship	Child	0 vs 1+ sessions	125	[2,16]	6.53 (3.09)	[1,12]	4.79 (1.80)	-1.85 (-2.29, -1.40)	<0.001	28%	0.35
		1–7 vs 8+ sessions	109	[2,16]	6.54 (3.18)	[1,12]	4.89 (1.88)	-1.76 (-2.12, -1.40)		27%	0.32

*P-value testing differences in effect size between the models.

#### Harsh parenting

Parents reported large effects for reduced harsh parenting from baseline to post-test (*f*^2^ = 0.41, *B* = -2.83, 95%CI [-3.13, -2.52]); 26% reduction, with male parents reporting greater reductions than female parents (*B* = -0.95, 95%CI [-1.28, -0.62]). Likewise, children reported large effects for reduced harsh parenting by fathers (*f*^2^ = 0.64, *B* = -4.55, 95%CI [-5.36, -3.73]) and reduced harsh parenting by mothers (*f*^2^ = 0.56, *B* = -4.63, 95%CI [-5.78, -3.47]). Parents who enrolled in the program (i.e., attended at least 1 session) reported greater reductions in harsh parenting than non-enrollees (*B* = -0.68, 95%CI [-1.23, -0.14]). However, those enrollees who attended more than 50% of the program reported similar reductions in harsh parenting as those who attended less (*B* = 0.03, 95% CI [-037, 0.43]). Caregivers aged 40 years and above reported higher reductions in harsh parenting than those aged below 40 years (overall: B = -0.32 (-0.66, 0.014); session attendees: B = -0.45 (-0.86, -0.05). Children did not report any significant associations between program participation and harsh parenting. However, more female children reported improved relationships with their male caregivers than the male children. Fig 2 shows the reduction in harsh parenting.

#### Dysfunctional partner relationships

Parents reported medium effects for reduced dysfunctional relationships between parents (*f*^2^ = 0.19, *B* = -2.89, 95%CI [-3.31, -2.47]), with marginal difference in improvement by sex with the male having a slightly higher improvement than the female. Children’s reports also indicated medium effects for reduction in dysfunctional relationships between parents (*f*^2^ = 0.28, *B* = -1.85, 95%CI [-2.29, -1.40]). Although there were no significant differences reported by parents who enrolled in the program and non-enrollees (*B* = -0.05, 95%CI [-0.84, 0.73), enrollees who attended more than 50% of the program reported greater reductions than those who attended less than half of the program (*B* = -0.74, 95%CI [-1.32, -0.16]). Fig 3, shows the reduction in dysfunctional relationships.

#### Secondary outcomes

Results for secondary outcomes are summarized in Table 4.

**Table 4:** Summary statistics, effect estimates, and effect sizes for secondary outcomes.

			Baseline		Post-test		Intervention effect			
Outcome	Report	N	Range	Mean (SD)	Range	Mean (SD)	Effect (95% CI)	p-value	% change	Cohen f^2^
Positive Parenting	Parent	483	[12,67]	47.38 (9.23)	[23,71]	57.17 (8.24)	9.82 (8.85, 10.78)	<0.001	21%	0.38
Positive Parenting—Mother	Child	209	[25,60]	47.75 (7.37)	[4,60]	53.42 (7.65)	5.70 (4.56, 6.83)	<0.001	12%	0.39
Positive Parenting—Father	Child	179	[1,53]	35.17 (9.56)	[1,54]	40.35 (10.31)	5.76 (3.49, 8.03)	<0.001	16%	0.28
Parent inefficacy	Parent	484	[1,13]	6.44 (2.24)	[3,13]	5.15 (1.56)	-1.27 (-1.50, -1.05)	<0.001	20%	0.17
Caregiver-child conflict	Parent	482	[5,15]	12.44 (2.12)	[5,15]	14.03 (1.43)	-1.58 (-1.79, -1.36)	<0.001	13%	0.29
Respectful child behavior	Parent	481	[5,16]	12.92 (2.29)	[8,16]	14.48 (1.63)	1.56 (1.32, 1.81)	<0.001	12%	0.21
Respectful child behavior	Child	212	[2,10]	4.80 (1.92)	[2,9]	4.00 (1.61)	0.80 (0.42, 1,17)	<0.001	17%	0.12
Child necessities	Parent	483	[4,24]	17.27 (3.93)	[5,24]	19.24 (3.97)	1.95 (1.56, 2.35)	<0.001	11%	0.08
Child necessities—Mother	Child	207	[4,16]	13.09 (2.48)	[5,16]	13.82 (2.23)	0.72 (0.37, 1.08)	<0.001	6%	0.08
Child necessities—Father	Child	176	[3,16]	11.61 (3.24)	[3,16]	12.95 (3.04)	1.58 (0.87, 2.30)	<0.001	14%	0.17
Co-parenting arrangements	Parent	419	[3,16]	11.24 (2.74)	[1,16]	13.01 (2.65)	1.82 (1.48, 2.16)	<0.001	16%	0.14
Partner involvement	Parent	416	[2,20]	13.89 (3.91)	[1,20]	15.91 (3.70)	2.10 (1.65, 2.55)	<0.001	15%	0.09
Community parenting	Parent	482	[3,11]	5.83 (2.08)	[2,12]	6.90 (2.68)	1.08 (0.83, 1.33)	<0.001	18%	0.09
Gender socialization	Parent	484	[19,47]	32.58 (4.16)	[21,49]	36.66 (4.70)	4.08 (3.61, 4.56)	<0.001	13%	0.24
Child development knowledge	Parent	484	[1,10]	6.83 (2.21)	[3,10]	8.73 (1.56)	1.89 (1.66, 2.13)	<0.001	28%	0.34

#### Positive parenting

Parents reported large effects for improved positive parenting (*f*^2^ = 0.38, *B* = 9.82, 95%CI [8.85, 10.78]); an increase of 21%, with greater improvements amongst female parents than male parents (*B* = 4.96, 95%CI [3.85, 6.07]). Children reported medium effects for improved positive parenting by fathers (*f*^2^ = 0.28, *B* = 5.76, 95%CI [3.49, 8.03]); 12% increase and mothers (*f*^2^ = 0.39, *B* = 5.70, 95%CI [4.56, 6.83]); 16% increase, with no differences between mothers and fathers.

#### Parent sense of inefficacy

Parents reported reduced sense of inefficacy in parenting (*f*^2^ = 0.17, *B* = -1.27, 95% CI [-1.50, -1.05]); 20% reduction, with mothers reporting greater effects than fathers (*B* = 0.59, 95% CI [0.33, 0.84]).

#### Respectful child behavior

Parents and children reported increased respectful child behavior at post-test; parents: *f*^2^ = 0.21, *B* = 1.56, 95%CI [1.32, 1.81], 12% increase; children: *f*^2^ 0.12, *B* = 0.80, 95%CI [0.42, 1.17], 17% increase. There were no differences by gender among both children and parent reports.

#### Caregiver-child conflict

Parents reported reduced caregiver-child conflict when comparing follow-up assessments with baseline scores (*f*^2^ = 0.29, *B* = -1.58, 95%CI [-1.79, -1.36]); 13% reduction, with fathers reporting less conflict than mothers (*B* = -0.71, 95%CI [-0.94, -0.48]).

#### Provision of child necessities

Parents reported a modest increased provision of child necessities at post-test (*f*^2^ = 0.08, *B* = 1.95, 95%CI [1.56, 2.35]); 11% increase, with fathers reporting higher levels than mothers (*B* = 0.83, 95%CI [0.28, 1.37]). Children reported increased child necessities from their fathers (*f*^2^ = 0.17, *B* = 1.58, 95%CI [0.87, 2.30]); 17% increase; but minimal increase from their mothers (*f*^2^ = 0.08, *B* = 0.72, 95%CI [0.37, 1.08]); 6% increase.

#### Co-parenting, parent involvement, and collective parenting

Parents reported improved co-parenting arrangements at post-test (*f*^2^ = 0.14, *B* = 1.82, 95%CI [1.48, 2.16]); 16% increase, with fathers reporting better arrangements than mothers (*B* = 1.24, 95%CI [0.86, 1.62]). There was improved partner involvement (*f*^2^ = 0.09, *B* = 2.10, 95%CI [1.65, 2.55]); 15% increase; fathers reporting more partner involvement than mothers (*B* = 2.55, 95%CI [1.99, 3.12]). Parents also reported increased community/collective parenting (*f*^2^ = 0.09, *B* = 1.08, 95%CI [0.83, 1.33]); 13% increase.

#### Attitudes towards gender socialization

Parents reported improved attitudes towards gender socialization of children at post-test (*f*^2^ = 0.24, *B* = 4.08, 95%CI [3.61, 4.56]); 13% increase, with no differences between fathers and mothers’ reports.

#### Knowledge of child development

Parents also reported improved knowledge of child development at post-test (*f*^2^ = 0.34, *B* = 1.89, 95%CI [1.66, 2.13]); 28% increase, with mothers reporting higher knowledge than fathers (*B* = 0.48, 95%CI [0.23, 0.73]).

## Discussion

We conducted a pre-post evaluation of a parenting program (PfR) to reduce both violence against children and intimate partner violence. We report outcomes relating to harsh parenting experience of physical violence, positive parenting, and dysfunctional relationships and conflict between spouses. The study combines data from both parents and their children, but being quasi-experimental causal attribution can only be tentative.

Our results suggest that the PfR program may be an efficacious intervention at improving parenting skills and reducing violence against children and spousal violence. Participating in the program was associated with higher reporting of non-violent discipline skills, with decreased use of physical violence as a first option, and increased use of alternative non-violent means by both female and male parents. This fits with findings from similar parenting programs in the region [27–30] and Uganda [31–33]. In our study older parents (40+ years) had higher reductions of harsh parenting than younger parents, perhaps suggesting the need for greater parenting training among younger parents.

Overall, reduced harsh parenting was reported by both parents, however, children’s reports indicated a larger effect for fathers. This suggests that the program is not only successful in engaging fathers but may also shift paternal behavior positively, which is often a challenging issue in similar interventions, and justifies the growing calls for greater and more active father involvement in caregiving. More girl children reported an overall improvement in relationships with fathers compared to boy children. In other settings it has been hypothesized that fathers tend to be less violent to their daughters than sons [34]. A Ugandan national survey found that fathers tended to be harsher to sons when administering corporal punishment [4].

With respect to dysfunctional relationship outcomes among spouses, we found slightly above moderate effect sizes, indicating the intervention might improve family relationships and reduce spousal violence. Overall, our results share a number of similarities with recent studies on this topic. The evaluation of the impact of SASA! on violence against women, [35] found large intervention effect on IPV, in particular women’s past year experience of physical violence, and improvements in couple communication. Qualitative studies of the change process in the SASA! intervention suggested that reflection around healthy relationships and communication skills learned through the program or community activists led to more positive interaction among couples, nurtured trust and respect between partners and reduced conflict and IPV [36].

Our findings provide further evidence of the greater benefit of targeting both female and male parents. The rate of recruitment and retention of men in our study was considerably higher than reported in many trials, and offers great promise on the viability of programs to promote male engagement, and could serve as a model for similar interventions. Strategies employed to achieve high male participation included presenting the program as targeting fathers as much as it does mothers, and explaining the expected benefits of greater male involvement to fathers in their own right. Our program deliberately recruited parental couples, and this increased the number of fathers, and its delivery structure involving formation of separate groups for fathers and mothers, initially, allowed them to participate in both single sex in the first half of the program and then completing the program in mixed sex sessions. Single sex sessions offered safe space for both sexes to initially explore gender sensitive parenting concerns and spousal relationships without fear of being judged, and fathers greatly welcomed this model.

In general, intimate partner violence in Sub-Saharan Africa is perpetrated by men. The PfR approach recognizes this fact, but rather than focusing much attention on demonizing men as perpetrators of violence and thereby risk discouraging from participating in the programme, it appeals to men’s positive motivation, and seeks to recruit them as allies in addressing family violence and poor parenting. The program’s theoretical perspective is sensitive, recognizes men as interested parties, and promotes their core aspiration to achieve family respectability by encouraging positive practices around co-existence, shared family values, and father’s roles, without reproducing existing predominant norms. This intervention’s approach, in particular involving spouses, was highly valued by couples, since conflictual perspectives on couple relationships, gender norms, and violence were discussed during the sessions in a respectful and participatory manner, led by a facilitator. This enabled couples to jointly reflect on the quality of their relationships and their individual roles in perpetrating conflict and violence. They found it easier to demand change by reminding each other of the program’s lessons and jointly exploring peaceful means to resolve differences without resorting to hostility as first option, thereby reducing incidences of IPV [20]. In contrast, although an evaluation of a fatherhood program in Northern Uganda [33] found significant, positive effects on couple communication, there were limited effects on attitudes justifying IPV and no effect on gender norms. In that program wives were not a primary target. Our findings add additional evidence by showing positive intervention effects on both men’s and women’s attitude towards equal gender socialisation. It would appear, therefore, that enrolling both women and men in a parenting program is critical to enhancing its effects on spousal relationships, family roles and gender norms [37].

It is widely recognized that changing gender norms, even at family level, is a long term engagement that requires instilling intervention values at community level [12,20,38]. As Ashburn et al have noted, additional engagement is needed with people influential at the community level to contribute more significantly to change in attitudes and norms and to sustain newly adopted behaviors. The *PfR* intervention could influence broader social and gender norms at a macro level by: a) its community-based, inclusive approach; b) delivery as a universal prevention programme rather than targeted; c) addressing the long-term, intergenerational transfer of gender socialising norms; d) scaling up the programme nationally pending results from a cluster RCT; and e) combining the programme with other population level norm changing interventions.

This study has particular strengths and limitations. *PfR* is a systematically developed, theoretically based, culturally-sensitive parenting program that aims to modify familial predictors of both poor parenting and intimate partner violence. It is closely aligned with the Ugandan Government’s National Strategy on parenting and families and both local NGOs and the Ministry of Gender, Labour and Social Development are keen to scale up the program. This study contributes to the current work of the Uganda Government’s National Parenting Agenda Consortium to standardize the parenting landscape by generating the much-needed country-specific evidence on effective parenting interventions and models. We conducted the study using validated tools, had a large sample size, and conducted robust statistical analyses that consider clustering within groups. A major contribution of the study is the success of *PfR* in recruiting and retaining fathers, not just primary caretakers (usually considered to be mothers). Details on the strategies employed to achieve high male participation have previously been published (20) which might inform similar interventions.

The study’s main limitation is that the quasi-experimental pre-post evaluation design made it impossible to consider and control for the secular changes that might have occurred during the study period [39]. Social desirability bias might have been exacerbated through the delivery of program messages over 16 sessions, leading participants to exaggerate their self-reported post-intervention data. There is also the possibility that given the sensitive nature of the topics involved, the participants may have tended to report outcomes that they perceive as more favorable or expected by the researchers. However, outcome data on parent-child and parent-parent relationships collected from children, who did not participate in the sessions, were likely to have greater validity since far less subject to social desirability bias. They too showed marked improvements in outcomes. Thus, the inclusion of both parents’ and children’s perspectives strengthens the findings and provides a multidimensional view of the program’s impact. Nevertheless, a controlled trial would have allowed stronger causal attribution.

## Conclusion

These results suggest that the *Parenting for Respectability* Program might be a feasible and effective model to simultaneously reduce VAC and IPV and, in the long term, gender-based violence in Uganda. Attending the program was associated with reduced harsh parenting, with more parents committed to adopting positive parenting and with better spousal relationships. Additionally, PfR contributes evidence on how to combine, practically, programming to address violence against women and VAC. A key contribution is that PfR successfully recruited high numbers of fathers and parental couples, and the process of its development provides valuable insights on steps to follow when developing a strong theoretically oriented and evidence-based home-grown parenting intervention (22). The results of this pre-post study suggest that the intervention warrants more rigorous evaluation through a randomized controlled trial. Further research is also needed to explore why the program had larger effects on older parents than younger ones, such as generational differences in parenting styles or receptivity to non-traditional parenting methods.

## Supporting information

S1 Checklist CONSORT 2010 checklist of information to include when reporting a randomised trial*. (DOC)

S1 File (DOCX)

S2 File (DOCX)

S3 File (DOCX)
