# Supplementary material for: The impact of the parenting for respectability programme on violent parenting and intimate partner relationships in Uganda: A pre-post study
Source: PLoS One. 2024 May 24;19(5):e0299927. doi: 10.1371/journal.pone.0299927 (PMC11125497; doi:10.1371/journal.pone.0299927)
Supplement: S1 File — (DOCX) [file pone.0299927.s002.docx]

| **Comment** | **Response** |
| --- | --- |
| Pease ensure that the author list and affiliations are correct on the title page of your manuscript, and that your author contributions, competing interests, and financial disclosure are correct as listed below. All of these sections will be indexed in PubMed and published by PLOS ONE as you have written them. Please email plosone@plos.org if any changes to this content need to be made.  Please see here for the full list and definition of contributor roles: <http://journals.plos.org/plosone/s/authorship#loc-author-contributions>  Please ensure that the Competing Interests and Financial Disclosure statements listed below are suitable for publication. These sections will be indexed in PubMed and published by PLOS ONE as you have written them. Please email plosone@plos.org if any changes to these statements need to be made.  Competing Interests:   Financial Disclosure: | The author list and affiliations have been checked and a few edits made.  Godfrey Siu^1,3,6^, Rebecca N Nsubuga^3^, Jamie M. Lachman^2,4,5^, Carol Namutebi^1^, Richard Sekiwunga^1^, Flavia Zalwango^3^, Julie Riddell^2^, Daniel Wight^2^  ^1^ Child Health and Development Centre, School of Medicine Makerere University College of Health Sciences  ^2^ MRC/CSO Social and Public Health Sciences Unit, University of Glasgow.  ^3^ MRC/UVRI & LSHTM Uganda Research Unit  ^4^ Department of Social Policy and Intervention, University of Oxford  ^5^University of Cape Town  ^6^Department of Global Health, LSHTM  **Declaration**  **Ethics approval and informed consent**  ***Ethics Statement***  The study was reviewed by the Research and Ethics Committee of the Uganda Virus Research Institute (UVRI) and thereafter approved by the Uganda National Council for Science and Technology (UNCST). Adult participants provided written informed consent following the administration of the information sheet that described the study purpose, procedures, data management, and their rights to voluntarily participate. Those who were not able to sign the consent form provided a thumb print. Parents consented for their children, but before being interviewed, children were asked to assent and were assured of protection in case they did not wish to participate or decided to end their participation during the course of the interview.  **Data availability**  **All data sets can be obtained from the principle investigator upon request.**  **Data availability**  All data can be obtained from the Corresponding Author, upon request  **Funding** This research was funded by Oak Foundation, the Sexual Violence Research Initiative, Bernard van Leer Foundation, and University of Glasgow. The content is solely the responsibility of the authors and does not represent the views of these funding agencies. None of the funders had a role in study design, data collection and analysis, decision to publish, or preparation of the manuscript.  **Competing Interest**  The authors report no conflict of interest.  **Authors’ contribution**  Godfrey Siu: Funding acquisition, conceptualization, supervision, formal analysis, writing – original draft, writing – review and editing  Rebecca N Nsubuga: Conceptualization, formal analysis, writing – original draft, writing – review and editing  Jamie M. Lachman: Formal analysis, writing – original draft, writing – review and editing  Carol Namutebi: Conceptualization, data collection, whiting – reviewing and editing  Richard Sekiwunga: Conceptualization, data collection, whiting – reviewing and editing  Flavia Zalwango: Conceptualization, data collection, whiting – reviewing and editing  Julie Riddell: Formal analysis, writing – original draft, writing – review and editing  Daniel Wight: Funding acquisition, conceptualization, formal analysis, writing – original draft, writing – review and editing  All authors gave final approval of the version to be published, have agreed on the journal to which the article has been submitted, and agree to be accountable for all aspects of the work.  **Acknowledgments** We acknowledge the support from the Research Assistants who diligently collected during both the baseline and endline. |
| 2. We note that you have not uploaded a completed CONSORT checklist. As detailed in PLOS Editorial Policy, a CONSORT checklist must be published alongside papers reporting clinical trials in the event of acceptance. In keeping with CONSORT reporting guidelines, please upload a completed CONSORT checklist (https://www.equator-network.org/reporting-guidelines/consort/). Please also amend your figure legends and captions as necessary. More information related to the relevant journal policies is available from: http://journals.plos.org/plosone/s/submission-guidelines#loc-clinical-trials. " | Thank you for this comment. However, our study was not a clinical trial. Nonetheless, we have attached a completed CONSORT checklist |
| 3. To prevent production delays, we recommend using the Author Formatting Checklist to confirm that your paper meets PLOS ONE's typesetting requirements for References, Tables, and Figures: http://journals.plos.org/plosone/s/file?id=c819/plos-one-author-formatting-checklist.docx.  This checklist is a reference tool for you; please do not upload the completed Author Formatting Checklist with your submission files. | We have double checked and noticed that our figures and tables meet the PLOS ONE typesetting requirements. |
| 4. To ensure your figures meet our technical requirements, please run each figure included in your submission files through the PACE tool: https://pacev2.apexcovantage.com/. PACE will assess whether your figures meet our technical requirements and will fix the figure(s) or identify any problem(s) that cannot be automatically fixed. It can also convert figures to TIFF format, resize, and rename figures to meet our naming conventions. To use PACE, first register as a user. Follow the instructions on the site for assessing and converting your figure files. If you experience any difficulty using this tool or have questions about any of the figures and/or images in your paper, please inform the journal office in your response letter. | Thank you. This has been done as advised. |
