# Supplementary material for: The impact of the parenting for respectability programme on violent parenting and intimate partner relationships in Uganda: A pre-post study
Source: PLoS One. 2024 May 24;19(5):e0299927. doi: 10.1371/journal.pone.0299927 (PMC11125497; doi:10.1371/journal.pone.0299927)
Supplement: S2 File — (DOCX) [file pone.0299927.s003.docx]

Responses to the impact of the Parenting for Respectability programme on violent parenting and intimate partner relationships in Uganda: a pre-post study manuscript.

| **S. No.** | **Comments** | **Response** |
| --- | --- | --- |
| 1. | 1. We note that figure/s 1, 2 and 3 have not been uploaded as separate files. Please ensure that all figures are uploaded to the submission system as a separate file in the file inventory. | Thankyou. We have been able to upload a separate “Figure File” in response to this requirement. |
| 2. | We note that you have included your Figures within the body of your manuscript. Please remove the Figures from the body of your manuscript and upload them as separate Figure files. | Thankyou. This has been addressed as advised. |
| 3. | Please ensure that each figure has a corresponding figure caption included in the main manuscript file. Guidelines for figure captions can be found at <http://journals.plos.org/plosone/s/figures#loc-captions> | Thank you. This has been done as guided. |
| 4 | Please ensure that each table has a corresponding table caption included in the main manuscript file. Guidelines for table captions can be found at <https://journals.plos.org/plosone/s/tables> | Thank you. This has been done as guided. |
| 5 | We note that you have included a Funding Statement within the body of your manuscript. Please note that declarative statements should not be included within the manuscript. Please ensure that the statement provided in the submission system is correct, and then remove the statement from your manuscript file. | Thank you. This has been done as guided. |
| 6 | Funding information for your study should not appear in the Acknowledgments section or other areas of your text, but must be entered into the online Editorial Manager in the Financial Disclosure field.  The Financial Disclosure in your published article will be derived from the information provided in the system.  Please remove any funding information from your manuscript, and ensure that the Financial Disclosure statement provided in the submission system is correct. | Thank you. This has been done as guided. |
| 7 | We note that you have included a Competing Interests Statement within the body of your manuscript. Please note that declarative statements should not be included within the manuscript. Please ensure that the statement provided in the submission system is correct, and then remove the statement from your manuscript file. | Thank you. This has been done as guided. |
| 8 | We note that you have included a Data Statement within the body of your manuscript. Please note that declarative statements should not be included within the manuscript. Please ensure that the statement provided in the submission system is correct, and then remove the statement from your manuscript file. | Thank you. This has been done as guided. |
| 9 | We note that you have included ethics statements in the [The study was reviewed by the Research and Ethics Committee of the Uganda Virus Research Institute (UVRI) and thereafter approved by the Uganda National Council for Science and Technology (UNCST). Adult participants provided written informed consent following the administration of the information sheet that described the study purpose, procedures, data management, and their rights to voluntarily participate. Those who were not able to sign the consent form provided a thumb print. Parents consented for their children, but before being interviewed, children were asked to assent and were assured of protection in case they did not wish to participate or decided to end their participation during the course of the interview.] section of your manuscript.  Please note that ethics statements should be included in the methods section of your manuscript only. Please ensure that the statements provided are correct and within your methods section. | Thank you. This has been done as guided. |
