# Supplementary material for: The impact of the parenting for respectability programme on violent parenting and intimate partner relationships in Uganda: A pre-post study
Source: PLoS One. 2024 May 24;19(5):e0299927. doi: 10.1371/journal.pone.0299927 (PMC11125497; doi:10.1371/journal.pone.0299927)
Supplement: S3 File — (DOCX) [file pone.0299927.s004.docx]

**Table 1: Summary of outcomes assessed at baseline and post-test**

| **Outcome** | **Source** | **No. of Items** | **Reliability^1^** |  |
| --- | --- | --- | --- | --- |
| Primary outcomes |  |  |  |  |
| Harsh parenting | Parent | 6 | 0.61 |  |
| Harsh parenting – male carer | Child | 8 | 0.72 |  |
| Harsh parenting – female carer | Child | 8 | 0.62 |  |
| Dysfunctional partner relationships | Parent | 9 | 0.78 |  |
| Dysfunctional partner relationships | Child | 4 | 0.85 |  |
| Secondary outcomes |  |  |  |  |
| Positive parenting | Parent | 18 | 0.84 |  |
| Positive parenting – male carer | Child | 14 | 0.91 |  |
| Positive parenting – female carer | Child | 15 | 0.84 |  |
| Parent sense of inefficacy | Parent | 4 | 0.45 |  |
| Caregiver-child conflict | Parent | 5 | 0.57 |  |
| Respectful child behavior | Parent | 4 | 0.60 |  |
| Respectful child behavior | Child | 3 | 0.68 |  |
| Provision of child necessities | Parent | 6 | 0.60 |  |
| Provision of child necessities – male carer | Child | 4 | 0.82 |  |
| Provision of child necessities – female carer | Child | 4 | 0.67 |  |
| Co-parenting arrangements | Parent | 4 | 0.53 |  |
| Partner involvement | Parent | 5 | 0.71 |  |
| Community parenting | Parent | 3 | 0.56 |  |
| Gender socialization – attitudes | Parent | 13 | 0.67 |  |
| Knowledge of child development | Parent | 5 | 0.30 |  |
| ^1^ Reliability at baseline based on Cronbach’s alpha coefficient | | | | |

**Table 2. Characteristics of the sample at baseline**

| **Characteristic** | **Freq/Mean** |
| --- | --- |
| **Parents (N=484)** |  |
| Sex, *n* (%)  Female  Male | 269 (56.0)  215 (44.0) |
| Age, M (SD) | 38.9 (10.9) |
| Marital status, *n* (%)  Married  Single/Widowed/Separated | 408 (84.5)  75 (15.5) |
| Education level  None/Incomplete Primary  Complete Primary and above | 205 (42.4)  278 (56.6) |
| Employment, *n* (%)  Farmer  Non-farmer | 249 (51.4)  235 (48.6) |
| **Children (N = 212)** |  |
| Sex, *n* (%)  Female  Male | 117 (55.2)  95 (44.8) |
| Age, M (SD) | 11.7 (1.5) |
| Relationship to parent, *n* (%)  Both parents  Single parent  Other/step/nonbiological | 124 (66.3)  47 (25.1)  16 (8.6) |
| Enrolled in school, *n* (%) | 201 (94.8) |
| **Household** |  |
| Respondent is only caregiver in household, *n* (%) | 62 (12.8) |
| Other parent lives in household (*N*=422), *n* (%) | 355 (84.1) |
| Number of children, M(SD) |  |
| Girls | 2.1 (1.7) |
| Boys | 2.2 (1.6) |
| Total | 4.3 (2.3) |
| Electricity in house, *n* (%) | 234 (49.1) |
| Piped water in compound, *n* (%) | 86 (17.8) |

**Table 3: Summary statistics, effect estimates, and effect sizes for primary outcomes**

|  |  |  |  | **Baseline** | | **Post-test** | |  |  |  |  |
| --- | --- | --- | --- | --- | --- | --- | --- | --- | --- | --- | --- |
| **Outcome** | **Model** | **Report** | **N** | **Range** | **Mean (SD)** | **Range** | **Mean (SD)** | **Effect (95% CI)** | **p-value** | **% change** | **Cohen f^2^** |
| Harsh Parenting | 0 vs 1+ sessions | Parent | 481 | [1,20] | 10.90 (2.96) | [5,17] | 8.08 (2.18) | -2.83 (-3.13, -2.52) | <0.001 | 26% | 0.41 |
|  | 1-7 vs 8+ sessions |  | 419 | [1,20] | 10.91 (2.98) | [5,17] | 7.93 (2.09) | -2.97 (-3.28, -2.66) |  | 27% | 0.47 |
| Harsh Mother | 0 vs 1+ sessions | Child | 171 | [9,28] | 17.17 (3.91) | [6,25] | 12.53 (3.55) | -4.63 (-5.78, -3.47) | <0.001 | 27% | 0.56 |
|  | 1-7 vs 8+ sessions |  | 151 | [9,28] | 16.99 (4.02) | [6,25] | 12.44 (3.49) | -4.55 (-5.72, -3.38) |  | 27% | 0.52 |
| Harsh Father | 0 vs 1+ sessions | Child | 142 | [8,27] | 15.77 (4.39) | [1,23] | 11.17 (3.44) | -4.55 (-5.36, -3.73) | <0.001 | 29% | 0.64 |
|  | 1-7 vs 8+ sessions |  | 124 | [8,26] | 15.57 (4.28) | [1,22] | 10.89 (3.21) | -4.40 (-5.23, -3.57) |  | 28% | 0.60 |
| Dysfunctional relationship | 0 vs 1+ sessions | Parent | 429 | [4,26] | 13.13 (3.68) | [0,27] | 10.25 (3.25) | -2.89 (-3.31, -2.47) | <0.001 | 22% | 0.19 |
|  | 1-7 vs 8+ sessions |  | 375 | [4.26] | 13.28 (3.75) | [0,27] | 10.04 (2.89) | -3.27 (-3.69, -2.85) |  | 25% | 0.26 |
| Dysfunctional relationship | 0 vs 1+ sessions | Child | 125 | [2,16] | 6.53 (3.09) | [1,12] | 4.79 (1.80) | -1.85 (-2.29, -1.40) | <0.001 | 28% | 0.35 |
|  | 1-7 vs 8+ sessions |  | 109 | [2,16] | 6.54 (3.18) | [1,12] | 4.89 (1.88) | -1.76 (-2.12, -1.40) |  | 27% | 0.32 |

**Table 4: Summary statistics, effect estimates, and effect sizes for secondary outcomes**

|  |  |  | **Baseline** | | **Post-test** | |  | **Intervention effect** | | | | |
| --- | --- | --- | --- | --- | --- | --- | --- | --- | --- | --- | --- | --- |
| **Outcome** | **Report** | **N** | **Range** | **Mean (SD)** | **Range** | **Mean (SD)** | **Effect**  **(95%CI)** | | **p-value** | **% change** | **Cohen f^2^** |  |
| Positive Parenting | Parent | 483 | [12,67] | 47.38 (9.23) | [23,71] | 57.17 (8.24) | 9.82 (8.85, 10.78) | | <0.001 | 21% | 0.38 |  |
| Positive Parenting - Mother | Child | 209 | [25,60] | 47.75 (7.37) | [4,60] | 53.42 (7.65) | 5.70 (4.56, 6.83) | | <0.001 | 12% | 0.39 |  |
| Positive Parenting - Father | Child | 179 | [1,53] | 35.17 (9.56) | [1,54] | 40.35 (10.31) | 5.76 (3.49, 8.03) | | <0.001 | 16% | 0.28 |  |
| Parent inefficacy | Parent | 484 | [1,13] | 6.44 (2.24) | [3,13] | 5.15 (1.56) | -1.27 (-1.50, -1.05) | | <0.001 | 20% | 0.17 |  |
| Caregiver-child conflict | Parent | 482 | [5,15] | 12.44 (2.12) | [5,15] | 14.03 (1.43) | -1.58 (-1.79, -1.36) | | <0.001 | 13% | 0.29 |  |
| Respectful child behavior | Parent | 481 | [5,16] | 12.92 (2.29) | [8,16] | 14.48 (1.63) | 1.56 (1.32, 1.81) | | <0.001 | 12% | 0.21 |  |
| Respectful child behavior | Child | 212 | [2,10] | 4.80 (1.92) | [2,9] | 4.00 (1.61) | 0.80 (0.42, 1,17) | | <0.001 | 17% | 0.12 |  |
| Child necessities | Parent | 483 | [4,24] | 17.27 (3.93) | [5,24] | 19.24 (3.97) | 1.95 (1.56, 2.35) | | <0.001 | 11% | 0.08 |  |
| Child necessities - Mother | Child | 207 | [4,16] | 13.09 (2.48) | [5,16] | 13.82 (2.23) | 0.72 (0.37, 1.08) | | <0.001 | 6% | 0.08 |  |
| Child necessities - Father | Child | 176 | [3,16] | 11.61 (3.24) | [3,16] | 12.95 (3.04) | 1.58 (0.87, 2.30) | | <0.001 | 14% | 0.17 |  |
| Co-parenting arrangements | Parent | 419 | [3,16] | 11.24 (2.74) | [1,16] | 13.01 (2.65) | 1.82 (1.48, 2.16) | | <0.001 | 16% | 0.14 |  |
| Partner involvement | Parent | 416 | [2,20] | 13.89 (3.91) | [1,20] | 15.91 (3.70) | 2.10 (1.65, 2.55) | | <0.001 | 15% | 0.09 |  |
| Community parenting | Parent | 482 | [3,11] | 5.83 (2.08) | [2,12] | 6.90 (2.68) | 1.08 (0.83, 1.33) | | <0.001 | 18% | 0.09 |  |
| Gender socialization | Parent | 484 | [19,47] | 32.58 (4.16) | [21,49] | 36.66 (4.70) | 4.08 (3.61, 4.56) | | <0.001 | 13% | 0.24 |  |
| Child development knowledge | Parent | 484 | [1,10] | 6.83 (2.21) | [3,10] | 8.73 (1.56) | 1.89 (1.66, 2.13) | | <0.001 | 28% | 0.34 |  |
