# Supplementary material for: The impact of the parenting for respectability programme on violent parenting and intimate partner relationships in Uganda: A pre-post study
Source: PLoS One. 2024 May 24;19(5):e0299927. doi: 10.1371/journal.pone.0299927 (PMC11125497; doi:10.1371/journal.pone.0299927)

Manuscript

Click here to access/download;Manuscript;PfR_Manuscript (1).docx

Impact of the *Parenting for Respectability* program on intimate partner violence and violence against children in Uganda: a pre-post study

Godfrey Siu[1, 3], Rebecca N Nsubuga[3], Jamie M. Lachman[2,4], Carol Namutebi[1], Richard Sekiwunga[1], Flavia Zalwango[3], Emmanuel Asher Ikwara[1], Julie Riddell[2], Daniel Wight[2]

[1] CHDC School of Medicine Makerere University College of Health Sciences

[2] MRC/CSO Social and Public Health Sciences Unit, University of Glasgow.

[3] MRC/UVRI & LSHTM Uganda Research Unit

[4] Department of Social Policy and Intervention, University of Oxford

Introduction

Violence against children (VAC) and Intimate partner violence (IPV) are connected and are major social, development and public health concerns[1, 2]. Globally it is estimated that approximately 30% ever-partnered women worldwide have experienced physical and/or sexual violence by an intimate partner at some point in their lives [3]. The prevalence of IPV among women in Uganda is very high[4]. A 2018 Uganda Demographic and Health Survey found that 36% of women had ever experienced partner physical violence, while 22% had ever experienced partner sexual violence [5]. Violence against children is extremely widespread globally, with approximately half of all children – one billion aged 2-17 years – reporting having experienced violence in the past year [6, 7]. The Uganda national VAC survey 2015 found that 59% girls and 68% boys had experienced physical violence in their childhood, and 35% girls and 17% boys had experienced sexual violence in their childhood [8]. Such violence in Uganda and most Sub-Saharan African countries is usually perpetrated by people known to children in their homes and

community [7, 9]. VAC and IPV are major causes of morbidity and mortality, they undermine the social functioning of the victims and their families, and have lifetime consequences for physical, sexual, reproductive and mental health [10, 11]. The prevention of both forms of violence would contribute to the achievement of many Sustainable Development Goals (SDGs) since they strain health systems, lower educational achievement and economic productivity, and undermine economic and social development, [12-14] and elimination of IPV is essential to Goal Five.

Several studies confirm the link between VAC and IPV [1, 15, 16], suggesting the need for an integrated approach to their prevention. A review by Geudes and colleagues identified six ways in which they are interrelated: they have many shared risk factors, starting in the family; social norms legitimize both and discourage children and women from seeking help; both often occur within the same household both can be transmitted across generations; they can have similar consequences across the lifespan, and finally, both intersect in adolescence, a time of heightened vulnerability to violence. [17].

Factors perpetuating IPV and VAC exist at multiple socio-ecological levels[18]. For IPV, familial level factors include having been abused as a child, having an absent or rejecting father, inter-partner conflict, and male control of wealth and decision-making[19]. Community level factors include women's isolation and male peer groups that legitimize men's violence. At the macro level IPV is associated with cultural norms that condone violence within the family, schools and community, establish rigid gender roles and link masculinity to toughness, male honour, dominance and ownership of women, and it thrives where policy, legislation and implementation of laws is weak [20].

The shared familial risk factors for IPV and VAC, and their origin in adverse early exposure, provide a great opportunity for early intervention through optimizing parenting influence[21]. An increasing number of parenting programs are therefore being implemented and

tested in LMICs to reduce VAC [22], and substantial evidence is currently shows that if delivered with fidelity and systematically, they can be effective in improving child outcomes [23, 24]. However, interventions directly addressing early prevention of both IPV and VAC in LMICS remain limited [25].

Furthermore, very few parenting programs in LMICs harness cultural drivers and pre-existing motivations to change behavior[26]. In sub-Saharan Africa little attention has been paid to one of the most important dimensions of parenthood for both mothers and fathers: the need to maintain the family's respectability, in large part achieved through the appropriate behavior of the children and their parents [27, 28]. This core motivation might be harnessed in the design of interventions to reduce spousal violence, modify negative parenting and encourage sensitive parenting, in order to reduce children's future risk of sexual, physical and/or emotional violence[29]. In Uganda, there is paucity of literature on parenting programs that deliberately recruit parental couples to complete both single and mixed sex sessions. We therefore designed a community-based parenting program, – *Parenting for Respectability* (*PfR*), – to address this gap in Uganda, and contribute evidence on how a parenting program can address both IPV and VAC[30]. Following careful formative evaluation [31] we conducted a pre-post study to establish whether there was sufficient evidence of effectiveness to warrant progression to a randomized controlled trial. This paper reports on this study.

Study objective

The objective was to generate initial quantitative and qualitative evidence of the effectiveness of the *PfR* program in modifying key outcome measures on parent-child relationships, relationships between partners and SGBV. Through this project we tested the program to provide further evidence of program acceptability, the plausibility of the measures, the intended mechanism of change and the effects of the intervention on key outcomes associated with

sexual and gender-based violence, in particular violence against children. The project was delivered in central Uganda, near Kampala city, in collaboration with SOS Children's Village, a local NGO working in Wakiso District.

Methods

Study design

We employed a pre-post study in fourteen villages from three sub-counties in the Wakiso District, Uganda. Kakiri and Ssisa sub-counties are basically rural, with subsistence farming the main economic activity, while Katabi is peri-urban with informal trade as the main activity. Six hundred and seventy (670) parents were approached during mobilization, out of whom 626 parents were interviewed using a questionnaire at baseline; and registered as potential participants in the group-based intervention. Baseline data were collected in 2017 from consenting parents and half of the number of parent participants provided a list of their children aged 8-15-year-old from whom one child was selected randomly for interviews, providing parent-child data. The *PfR* sessions were then delivered to the parents for four months, and follow-up data collected three months post *PfR* completion in 2018.

Participants

Parents were first informed about the PfR Programme through local leaders, who went door-to-door, with support from the project team. Those interested in learning more were invited to a group meeting at the community centre, to receive study information. Anybody who identified as a parent, either biological parents or caregivers, was eligible as long as they were currently parenting children aged 0 – 17 years. The programme particularly emphasized the recruitment of fathers and parental couples. Once groups were recruited, the programme was then delivered once a week for 15 weeks by local facilitators selected from amongst the parents.

Data collection, management, and ethical procedures

Trained and supervised research assistants conducted written informed consent procedures using consent forms approved by both the ethics review boards of Uganda Virus Research Institute (GC/127/18/02/584) and Uganda National Council for Science and Technology (SS 4228). The questionnaires were translated into Luganda, the most widely spoken language in the area, and data collection took place between June and July, 2017 for baseline study and between March and May 2018 for post intervention study. Data was entered into an OpenClinica software database with data cleaning conducted using Stata 15. The study protocol was approved by the ethics review boards of Uganda Virus Research Institute (GC/127/18/02/584) and Uganda National Council for Science and Technology (SS 4228) and at the University of Glasgow.

The *Parenting for Respectability* Program

Parenting for respectability program (PfR) developed over five years follows the Six Steps for Quality Intervention Development (6SQuID) model [32]. Formative evaluation was initially conducted for over two years (2014-2016) in three villages involving three groups in Wakiso District. The parenting for respectability program consists of sixteen sessions that are inform of a program manual starting with nine single sex sessions followed by seven mixed sessions that are delivered once a week by two local facilitators who receive one week's training[26]. The program draws on parents' pre-existing motivation to maintain respectability, largely achieved through children's good behavior and respect for elders, and builds on parents' existing skills and experience. The program addresses four familial processes associated with GBV and VAC: poor parental bonding and child attachment; harsh parenting; inequitable socialization by gender and parental conflict[30]. A particular goal is to involve fathers, whom most parenting programs find hard to recruit, and the first nine sessions are delivered in single-sex groups, as a way to encourage

more fathers to attend. The intervention's rationale, program theory and formative evaluation are described elsewhere [33].

Measures

Demographic outcomes

Demographic data for parents and children were collected at baseline. Family characteristics included parent/child sex and age; caregiver relationship to child; type of caregivers in each household; child school attendance; and household size, type of household, income, and hunger (e.g., frequency in past month that family experienced went to bed hungry).

Primary outcomes

Primary outcomes for this study were changes in level of **harsh parenting** (parent- and child-report) and **dysfunctional partner relationships** (parent- and child-report). Parent-report (6 items) and child-report (8 items) of **harsh parenting** was based on the frequency of verbal and physical abuse in the past month (e.g., "How often do you hit your child with a stick or other objects when he/she has done something wrong?") with items ranging from 1 (never) to 4 (often). Children reported on both their female and male caregivers which were analysed separately. Parent-report (9 items) and child-report (4 items) of **dysfunctional partner relationships** was based on the frequency of verbal and physical conflict between male and female partners (e.g., "How often is there serious anger or hostility between you and your partner") with each item ranging from 1 (not at all) to 4 (more than once a week)[30].

Secondary outcomes

Secondary outcomes included positive parenting (parent-report: 18 items; child-report: 15-items), respectful child behavior (parent/child-report: 4 items), care-child conflict (parent-report: 5 items), parent self-inefficacy (parent-report: 4 items), provision of necessities as proxy for child neglect (parent/child-report: 6/14 items), co-parenting (parent-report: 4 items), partner

involvement in caregiving (parent-report: 5 items), and community/collective parenting (parent-report: 3 items), attitudes towards gender socialization (parent-report: 13 items), and knowledge of child development (parent-report: 5 items).

All measurements were previously validated in Wakiso during a formative evaluation study in 2018, with psychometric properties being examined further using baseline assessments.

INSERT TABLE 1 HERE

Process outcomes

Participation data were collected by program facilitators using attendance registers. Enrolment referred to whether participants attended at least one group session out of the total number of sessions. Attendance rates were calculated based on enrollees only [34].

Sample size calculations

A total of 645 parents and 261 of their children participated in the baseline survey. We initially recruited 400 parents and 182 children for the study and later recruited a further 245 parents and 79 children. From the first recruitment we experienced drop out of 161 parents from the program after minimal involvement due, largely, to the unmet expectations for material benefits despite this having been explained at recruitment. With 645 parents; assuming a 5% level of significance and standard deviation of the change in an outcome of as high as 1 we would be able to detect an effect of *PfR* as low as 0.11 with 80% power. We used the sample size formula of Rosner for before-and-after studies based on a paired T-test [35].

Data analysis

Frequencies for several socio-demographic and risk factors were calculated to assess their distribution. We then conducted confirmatory factor analyses and computed outcome scores with Cronbach's Alphas [36]. Linear mixed models accounting for random effects on the village and individual level were used to examine changes from baseline to post-test on primary and secondary

outcomes. Fixed factors included assessment time point, child/caregiver gender, and caregiver age, allowing for analyses of differential effects by gender and age as well. Full information maximum likelihood estimation method was used to account for missing data. To assess relevance of outcomes, tests for precision of effect sizes were undertaken (i.e., 95% confidence intervals did not overlap zero), as well as the direction and magnitude of bias corrected Cohen's f^2 effect sizes. An effect size of 0.02 was considered small, 0.15 moderate, and 0.35 or greater, large [37]. Finally, we examined the impact of program enrolment and attendance on primary outcomes by adding either a dichotomous variable for enrolment (yes/no) or for those who attended either more or less than 50% of the 15 sessions to the statistical models.

The scales were previously validated in Wakiso during the formative evaluation, with psychometric properties being examined further using baseline assessments. To examine the pre-post change on primary and secondary outcomes, baseline and post-test scores are compared using ANCOVA, incorporating relevant continuous covariates, or chi-square tests for categorical variables. The threshold for statistical significance was set at the $p < 0.05$ level (2-tailed).

Results

Socio-demographic characteristics of the sample at baseline

A total of 484 parents (*n*=269 mothers, 215 fathers) and 212 children (*n*=117 girls, 95 boys) completed the 3-months post-test survey. Distributions of the socio-demographic and risk factors are presented in Table 2. Caregiver age ranged between 18 and 82 years with a mean of 39, SD (10.9). Children were between 8 and 15 years, with 65% below 12 years. Enrolment rate was 87% (421/484) parents attending at least 8 of the 15 *PfR* sessions; while 15 (3%) parents attended all

the sessions. Out of the 212 children, 175 (82%) were linked to a household from the parents' database; and 154 (88%) had a caregiver who attend at least one session.

Primary outcomes

Results for primary outcomes are summarized in Table 3 and Figures 1-4.

Harsh parenting

Parents reported large effects for reduced harsh parenting from baseline to post-test (f^2=0.41, *B*=-2.83, 95%CI [-3.13, -2.52]); 26% reduction, with male parents reporting greater reductions than female parents (*B*=-0.95, 95%CI [-1.28, -0.62]). Likewise, children reported large effects for reduced harsh parenting by fathers (f^2=0.64, *B* =-4.55, 95%CI [-5.36, -3.73]) and reduced harsh parenting by mothers (f^2=0.56, *B*=-4.63, 95%CI [-5.78, -3.47]). Parents who enrolled in the program (i.e., attended at least 1 session) reported greater reductions in harsh parenting than non-enrollees (*B*=-0.68, 95%CI [-1.23, -0.14]). However, those enrollees who attended more than 50% of the program reported similar reductions in harsh parenting as those who attended less (*B*=0.03, 95% CI [-037, 0.43]). Caregivers aged 40 years and above reported higher reductions in harsh parenting than those aged below 40 years (overall: B=-0.32 (-0.66, 0.014); session attendees: B=-0.45 (-0.86, -0.05)). Children did not report any significant associations between program participation and harsh parenting. However, more female children reported improved relationships with their male caregivers than the male children.

Dysfunctional partner relationships

Parents reported medium effects for reduced dysfunctional relationships between parents (f^2=0.19, *B*=-2.89, 95%CI [-3.31, -2.47]), with marginal difference in improvement by sex with the male having a slightly higher improvement than the female. Children's reports also indicated medium effects for reduction in dysfunctional relationships between parents (f^2=0.28, *B*=-1.85,

95%CI [-2.29, -1.40]; Figure 3). Although there were no significant differences reported by parents who enrolled in the program and non-enrollees (*B*=-0.05, 95%CI [-0.84, 0.73), enrollees who attended more than 50% of the program reported greater reductions than those who attended less half of the program (*B*=-0.74, 95%CI [-1.32, -0.16]).

Secondary outcomes

Results for secondary outcomes are summarized in Table 4.

Positive parenting

Parents reported large effects for improved positive parenting (f^2=0.38, *B*=9.82, 95%CI [8.85, 10.78]); an increase of 21%, with greater improvements amongst female parents than male parents (*B*=4.96, 95%CI [3.85, 6.07]). Children reported medium effects for improved positive parenting by fathers (f^2=0.28, *B*=5.76, 95%CI [3.49, 8.03]); 12% increase and mothers (f^2=0.39, *B*=5.70, 95%CI [4.56, 6.83]); 16% increase, with no differences between mothers and fathers.

Parent sense of inefficacy

Parents reported reduced sense of inefficacy in parenting (f^2 = 0.17, *B*=-1.27, 95% CI [-1.50, -1.05]); 20% reduction, with mothers reporting greater effects than fathers (*B*=0.59, 95% CI [0.33, 0.84]).

Respectful child behavior

Parents and children reported increased respectful child behavior at post-test; parents: f^2=0.21, *B*=1.56, 95%CI [1.32, 1.81], 12% increase; children: f^2 0.12, *B*=0.80, 95%CI [0.42, 1.17], 17% increase. There were no differences by gender among both children and parent reports.

Caregiver-child conflict

Parents reported reduced caregiver-child conflict when comparing follow-up assessments with baseline scores (f^2=0.29, *B*=-1.58, 95%CI [-1.79, -1.36]); 13% reduction, with fathers reporting less conflict than mothers (*B*=-0.71, 95%CI [-0.94, -0.48]).

Provision of child necessities

Parents reported a modest increased provision of child necessities at post-test (f^2=0.08, *B*=1.95, 95%CI [1.56, 2.35]); 11% increase, with fathers reporting higher levels than mothers (*B*=0.83, 95%CI [0.28, 1.37]). Children reported increased child necessities from their fathers (f^2=0.17, *B*=1.58, 95%CI [0.87, 2.30]); 17% increase; but minimal increase from their mothers (f^2=0.08, *B*=0.72, 95%CI [0.37, 1.08]); 6% increase.

Co-parenting, parent involvement, and collective parenting

Parents reported improved co-parenting arrangements at post-test (f^2=0.14, *B*=1.82, 95%CI [1.48, 2.16]); 16% increase, with fathers reporting better arrangements than mothers (*B*=1.24, 95%CI [0.86, 1.62]). There was improved partner involvement (f^2=0.09, *B*=2.10, 95%CI [1.65, 2.55]); 15% increase; fathers reporting more partner involvement than mothers (*B*=2.55, 95%CI [1.99, 3.12]). Parents also reported increased community/collective parenting (f^2=0.09, *B*=1.08, 95%CI [0.83, 1.33]); 13% increase.

Attitudes towards gender socialization

Parents reported improved attitudes towards gender socialization of children at post-test (f^2=0.24, *B*=4.08, 95%CI [3.61, 4.56]); 13% increase, with no differences between fathers and mothers' reports.

Knowledge of child development

Parents also reported improved knowledge of child development at post-test (f^2=0.34, *B*=1.89, 95%CI [1.66, 2.13]); 28% increase, with mothers reporting higher knowledge than fathers (*B*=0.48, 95%CI [0.23, 0.73]).

Discussion

We employed a pre-post evaluation of a parenting program (PfR) to reduce both violence against children and intimate partner violence. We report outcomes on parent-child, harsh parenting and experience of physical violence, positive parenting, and dysfunctional relationships and conflict between spouses. This study combines data from both the parents and their children.

Our results suggest that the PfR program may be an efficacious intervention at improving parenting skills and reducing violence against children and spousal violence. We found that the program resulted in reported non-violent discipline skills, with decreased use of physical violence as a first option, and increased use of alternative non-violent means by both female and male parents. This confirms findings from similar parenting programs in the region [38-41] and Uganda [42-44]. In our study older parents (40+ years) had higher reductions of harsh parenting, than younger parents, perhaps suggesting the need for greater parenting training among younger parents.

Overall, there was reduced harsh parenting by both parents, however, children's reports indicated a larger effect by fathers, justifying the growing calls for father involvement. More girl children reported an overall improvement in relationships with a male parent compared to boy children. In other settings it has been hypothesized that fathers tend to be less violent to their daughters than sons [45]. A Ugandan national survey found that fathers tended to be harsher to sons when administering corporal punishment [8].

With respect to dysfunction relationship outcomes among spouses, we found slightly above moderate effect sizes, indicating the intervention improved family relationships and reduced spousal violence. Overall, our results share a number of similarities with recent studies on this

topic. In the evaluation of the impact of SASA! on violence against women, [46] found large intervention effect on IPV, in particular women's past year experience of physical violence, and improvements in couple communication. Qualitative studies of the change process in the SASA! intervention suggested that reflection around healthy relationships and communication skills learned through the program or community activists led to more positive interaction among couples, nurtured trust and respect between partners and reduced conflict and IPV [47]. Our findings provide further evidence of the greater benefit of targeting both female and male parents. Our program deliberately recruited parental couples and the structure of delivery allowed them to participate in both single sex (men only and women's only sessions) and mixed sex groups. Home practice activities encouraged parental couples to continue to reflect on session activities and try new skills at home. This intervention approach, in particular, involving spouses, was highly valued by couples, since conflictual perspectives on couple relationships, gender norms, and violence were discussed during the sessions in a respectful and participatory manner, led by a facilitator. This enabled couples to jointly reflect on the quality of their relationship and on one's own role in perpetrating conflict and violence, and they found it easier to demand change by reminding each other of the lessons from the program and jointly exploring peaceful means to resolve differences without resorting to hostility as first option, thereby reducing incidences of IPV [31].

In contrast, although an evaluation of a parenting program designed for fathers in Northern Uganda Ashburn et al. [44] found significant, positive effects of the intervention on couple communication, there was limited effects on attitudes justifying IPV and no effect on gender norms. In that program wives/ women were not a primary target. Thus it would appear that enrolling both women and men in a parenting program is a critical element that enhances the effects of a parenting model on both spousal relationships, family roles and gender norms [48]. Nonetheless, it is widely recognized that changing gender norms, even at family level, is a long

term engagement that requires instilling intervention values at community level [17, 31, 49]. As Ashburn et al have noted, there is need for additional engagement with other influential individuals at community level to contribute more significantly to change in attitudes and norms and sustain new behavior adapted.

This study has particular strengths and limitations. The *PfR* model is a systematically developed, theoretically based, culturally-sensitive parenting program that aims to modify familial predictors of gender-based violence. It is closely aligned with the Ugandan Government's National Strategy on parenting and families and both local NGOs, and the Ministry of Gender, Labour and Social Development are keen to scale up the program[50]. We conducted the study using validated tools, and conducted robust analysis, and complemented it qualitative assessments. The main limitation is with respect to the weakness of the pre-post evaluation design, which made it impossible to consider and control for the secular changes that might have occurred during the study period [51]. The intervention participants may have exaggerated the self-reported post-intervention data, thereby introducing the social desirability bias. A comparative study would have better accounted for cause-effect relationship. However, including children, who were not directly targeted by the intervention, to provide parent-child dyadic data both at pre-post intervention, may have mitigated limitations associated with the validity of the pre-post design.

Conclusion

These results suggest that *PfR* program might be a feasible and effective way to simultaneously reduce VAC and improve IPV and, in the long term, gender-based violence in Uganda. The program reduces parents' use of harsh parenting, with more parents committed to adopting positive parenting and contributes to better spousal relationships. Additionally, PfR contributes evidence on how to practically bring together programming addressing violence

against women and violence against children. A key contribution is that PfR successfully recruited high numbers of fathers, and parental couples, and the process of its development provides valuable insights on steps to follow while developing a strong theoretically oriented and evidence-based home-grown parenting intervention (Wight et al 2022). The results of this pre-post intervention study suggest that the intervention warrants further rigorous evaluation.

References

1. Carlson, C., et al., *Violence against children and intimate partner violence against women: overlap and common contributing factors among caregiver-adolescent dyads.* BMC Public Health, 2020. **20**(1): p. 1-13.
2. Pearson, I., et al., *The co-occurrence of intimate partner violence and violence against children: A systematic review on associated factors in low-and middle-income countries.* Trauma, Violence, & Abuse, 2022: p. 15248380221082943.
3. WHO, *Global Status Report on Violence Prevention* 2014, World Health Organisation: Geneva.
4. Young, C.R., et al., *Prevalence and correlates of physical and sexual intimate partner violence among women living with HIV in Uganda.* PloS one, 2018. **13**(8): p. e0202992.
5. Uganda Bureau of Statistics (UBOS) and ICF, *Uganda Demographic and Health Survey 2016*. 2018: Kampala, Uganda and Rockville, Maryland, USA: UBOS and ICF.
6. Hillis, S., et al., *Global Prevalence of Past-year Violence Against Children: A Systematic Review and Minimum Estimates.* Pediatrics, 2016. **137**(3): p. e20154079.
7. Ravi, S. and R. Ahluwalia, *What explains childhood violence? Micro correlates from VACS surveys.* Psychology, Health & Medicine, 2017. **22**(sup1): p. 17-30.

8. Ministry of Gender Labour and Social Development, *Violence against Children in Uganda: Findings from a National Survey, 2015. Kampala, Uganda: UNICEF, 2015.*, M.o. Gender, Editor. 2015: Kampala.
9. The Republic of Uganda, *Uganda Violence against Children Survey: Findings from a National Survey 2015*, M.o.G.L.a.S. Development, Editor. 2017, Ministry of Gender, Labour and Social Development: Kampala.
10. World Health Organization, *Preventing violence: Evaluating outcomes of parenting programmes*. 2013, WHO Press: Geneva.
11. Knaul, F.M., F. Bustreo, and R. Horton, *Countering the pandemic of gender-based violence and maltreatment of young people: The Lancet Commission.* Lancet, 2020. **395**(10218): p. 98-99.
12. Hillis, S.D., J.A. Mercy, and J.R. Saul, *The enduring impact of violence against children.* Psychology, Health & Medicine, 2017. **22**(4): p. 393-405.
13. Pereznieto, P., et al., *The Cost and Economic Impact of Violence Against Children,* O.D. Institute., Editor. 2014: London.
14. Hoeffler, A., *Violence against Children: A Critical Issue for Development*. 2017, Springer.
15. Chiang, L., et al., *Cycle of violence among young Kenyan women: The link between childhood violence and adult physical intimate partner violence in a population-based survey.* Child abuse & neglect, 2018. **84**: p. 45-52.
16. Namy, S., et al., *Towards a feminist understanding of intersecting violence against women and children in the family.* Social Science & Medicine, 2017. **184**: p. 40-48.
17. Guedes, A., et al., *Bridging the gaps: a global review of intersections of violence against women and violence against children.* Global health action, 2016. **9**(1): p. 31516.

18. Khuzwayo, N. and M. Taylor, *Exploring the socio-ecological levels for prevention of sexual risk behaviours of the youth in uMgungundlovu District Municipality, KwaZulu-Natal.* African Journal of Primary Health Care and Family Medicine, 2018. **10**(1): p. 1-8.

19. Khaemba, M.E.A., *Childhood exposure to inter-parental violence as a risk factor for intimate partner violence–a study on women in resource-limited settings in Nairobi, Kenya*. 2014, University of Nairobi.

20. Heise, L.L. and A. Kotsadam, *Cross-national and multilevel correlates of partner violence: an analysis of data from population-based surveys.* The Lancet Global Health, 2015. **3**(6): p. e332-e340.

21. Artz, S., et al., *A comprehensive review of the literature on the impact of exposure to intimate partner violence on children and youth.* 2014.

22. Gardner, F., et al., *Parenting for preventing risk of child maltreatment in low- and middle-income countries: Updated systematic review and meta-analysis.* forthcoming.

23. Singla, D.R., E. Kumbakumba, and F.E. Aboud, *Effects of a parenting intervention to address maternal psychological wellbeing and child development and growth in rural Uganda: A community-based, cluster-randomised trial.* The Lancet Global Health, 2015. **3**(8): p. e458-e469.

24. Shenderovich, Y., et al., *Delivering a Parenting Program in South Africa: The Impact of Implementation on Outcomes.* Journal of Child and Family Studies, 2019. **28**(4): p. 1005-1017.

25. Bacchus, L.J., et al., *Exploring opportunities for coordinated responses to intimate partner violence and child maltreatment in low and middle income countries: a scoping review.* Psychology, Health & Medicine, 2017. **22**(sup1): p. 135-165.

26. Siu, G.E., et al., *Men’s involvement in a parenting programme to reduce child maltreatment and gender-based violence: Formative evaluation in Uganda.* The European Journal of Development Research, 2017. **29**: p. 1017-1037.

27. Siu, G.E., J. Seeley, and D. Wight, *Dividuality, masculine respectability and reputation: how masculinity affects men's uptake of HIV treatment in rural eastern Uganda.* Soc Sci Med, 2013. **89**: p. 45-52.

28. Wamoyi, J. and D. Wight, *'Mum never loved me.' How structural factors influence adolescent sexual and reproductive health through parent-child connectedness: a qualitative study in rural Tanzania.* African journal of AIDS research : AJAR, 2014. **13**(2): p. 169-178.

29. Siua, G.E., et al., *Special Issue Article Men’s Involvement in a Parenting Programme to Reduce Child Maltreatment and Gender-Based Violence: Formative Evaluation in Uganda.* The European Journal of Development Research, 2017. **29**(5): p. 1017-1037.

30. Wight, D., et al., *A Ugandan parenting programme to prevent gender-based violence: description and formative evaluation.* Research on social work practice, 2022. **32**(4): p. 448-464.

31. Siu, G.E., et al., *Men’s involvement in a parenting programme to reduce child maltreatment and gender-based violence: Formative evaluation in Uganda.* The European Journal of Development Research, 2017. **29**(5): p. 1017-1037.

32. Wight, D., et al., *Six steps in quality intervention development (6SQuID).* Journal of Epidemiology and Community Health, 2016. **70**(5): p. 520-525.

33. Wight, D., et al., *A Ugandan Parenting Programme to Prevent Gender-Based Violence: Description and Formative Evaluation.* Research on Social Work Practice, 2022: p. 10497315211056246.

34. Baker, C.N., D.H. Arnold, and S. Meagher, *Enrollment and attendance in a parent training prevention program for conduct problems.* Prevention Science, 2011. **12**(2): p. 126-138.

35. Rosner, B., *Fundamentals of Biostatistics*. 4th ed. 1995: Duxbury Press.

36. Tavakol, M. and R. Dennick, *Making sense of Cronbach's alpha.* International Journal of Medical Education, 2011. **2**: p. 53-55.

37. Cohen, J., *Statistical power analysis for the behavioral sciences*. 1988, Hillsdale, NJ: Erlbaum.

38. Cluver, L.D., et al., *Parenting for Lifelong Health: a pragmatic cluster randomised controlled trial of a non-commercialised parenting programme for adolescents and their families in South Africa.* BMJ Global Health, 2018. **3**(1): p. e000539.

39. Ismayilova, L. and L. Karimli, *Harsh parenting and violence against children: A trial with ultrapoor families in Francophone West Africa.* Journal of Clinical Child & Adolescent Psychology, 2020. **49**(1): p. 18-35.

40. Cluver, L.D., et al., *Development of a parenting support program to prevent abuse of adolescents in South Africa: Findings from a pilot pre-post study.* Research on social work practice, 2017. **27**(7): p. 758-766.

41. Lachman, J.M., et al., *Randomized controlled trial of a parenting program to reduce the risk of child maltreatment in South Africa.* Child Abuse Negl, 2017. **72**: p. 338-351.

42. Kyegombe, N., et al., *What is the potential for interventions designed to prevent violence against women to reduce children's exposure to violence? Findings from the SASA! study, Kampala, Uganda.* Child Abuse & Neglect, 2015. **50**: p. 128-140.

43. Singla, D.R., E. Kumbakumba, and F.E. Aboud, *Effects of a parenting intervention to address both maternal psychological wellbeing and child development and growth in*

rural Uganda: a community-based, cluster randomised trial (vol 3, pg e458, 2015). LANCET GLOBAL HEALTH, 2016. **4**(1): p. E28-E28.

44. Ashburn, K., et al., *Evaluation of the responsible, engaged, and loving (REAL) fathers initiative on physical child punishment and intimate partner violence in Northern Uganda.* Prevention science, 2017. **18**(7): p. 854-864.

45. Cui, N., et al., *Does the gender of parent or child matter in child maltreatment in China?* Child abuse & neglect, 2016. **54**: p. 1-9.

46. Abramsky, T., et al., *The impact of SASA!, a community mobilisation intervention, on women's experiences of intimate partner violence: secondary findings from a cluster randomised trial in Kampala, Uganda.* J Epidemiol Community Health, 2016. **70**(8): p. 818-825.

47. Starmann, E., et al., *Exploring couples' processes of change in the context of SASA!, a violence against women and HIV prevention intervention in Uganda.* Prevention science, 2017. **18**(2): p. 233-244.

48. Doyle, K., et al., *Transforming gender roles in domestic and caregiving work: preliminary findings from engaging fathers in maternal, newborn, and child health in Rwanda.* Gender & Development, 2014. **22**(3): p. 515-531.

49. Starmann, E., et al., *Examining diffusion to understand the how of SASA!, a violence against women and HIV prevention intervention in Uganda.* BMC public health, 2018. **18**(1): p. 616.

50. Yoshikawa, H., et al., *Toward high‐ quality early childhood development programs and policies at national scale: Directions for research in global contexts.* Social Policy Report, 2018. **31**(1): p. 1-36.

51. Eccles, M., et al., *Research designs for studies evaluating the effectiveness of change and improvement strategies.* Quality and Safety in Health Care, 2003. **12**(1): p. 47-52.

Figure

Click here to access/download;Figure;Figure 1 Participant flow diagram.docx

Figure 1. Participant flow diagram

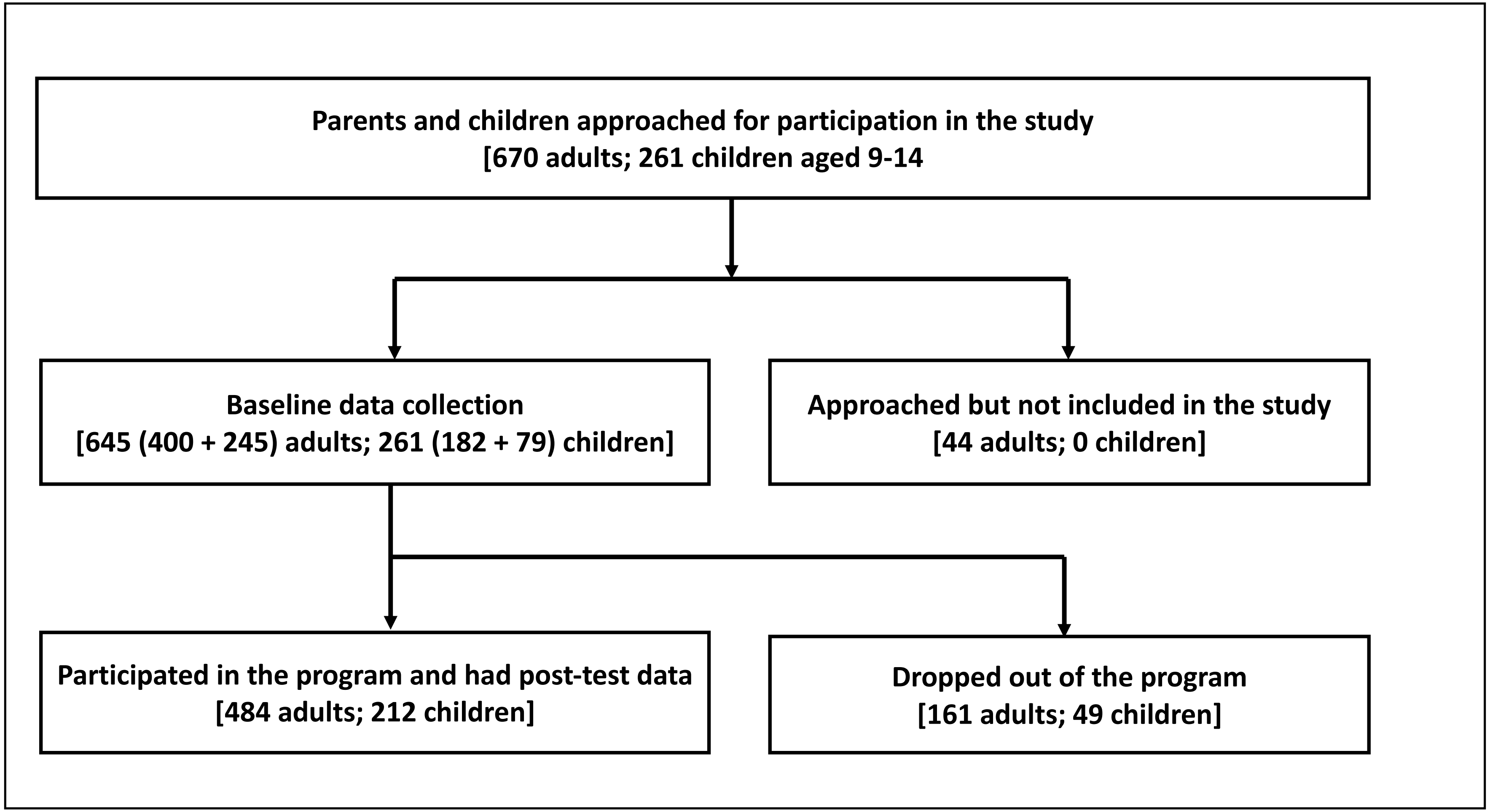

Figure Click here to access/download;Figure;Figure 2 Changes adjusted harsh parenting (1).docx

Figure 2. Changes (adjusted) from baseline to post-test for harsh parenting

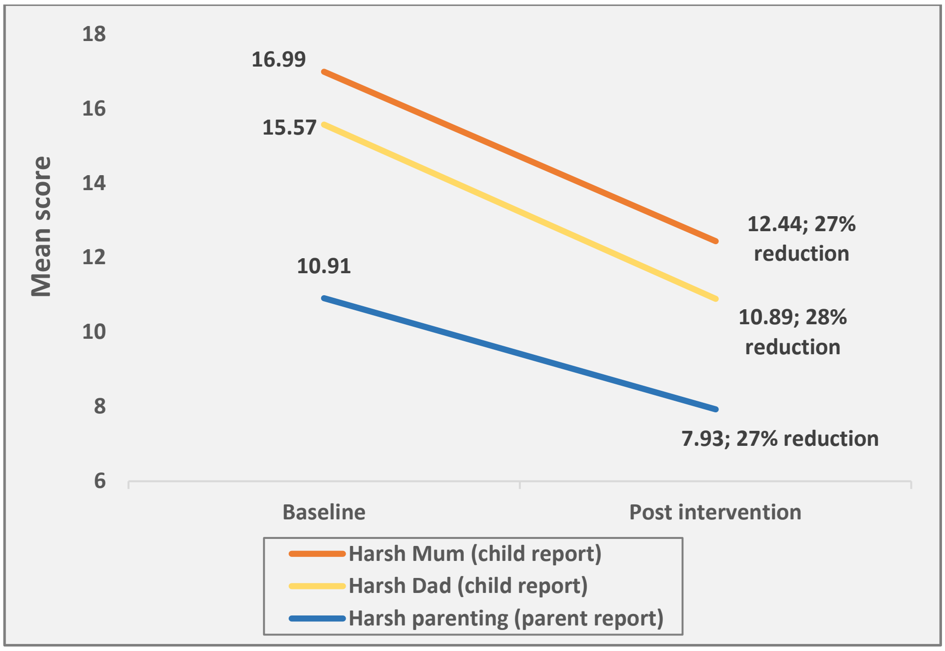

Click here to access/download;Figure;Figure 3 Dyfunctional relationships.docx

Figure 3: Changes (adjusted) from baseline to post-test for dysfunctional relationships

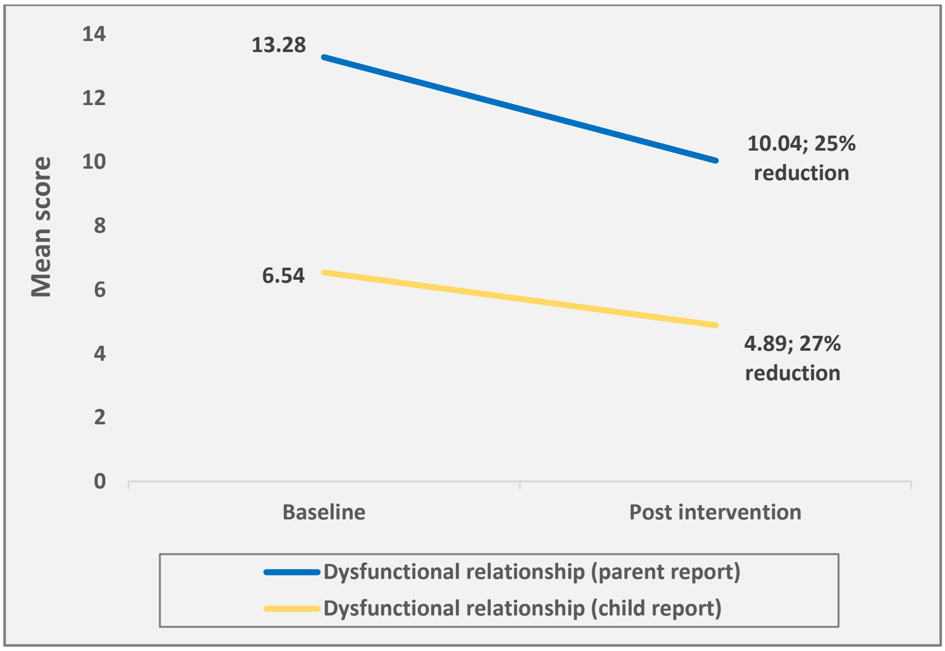

Supplement: Attachment — Submitted filename: Specific Comments.pdf [file pone.0299927.s005.pdf]
