# Supplementary material for: The impact of the parenting for respectability programme on violent parenting and intimate partner relationships in Uganda: A pre-post study
Source: PLoS One. 2024 May 24;19(5):e0299927. doi: 10.1371/journal.pone.0299927 (PMC11125497; doi:10.1371/journal.pone.0299927)
Supplement: Attachment — Submitted filename: Response to Reviewers letter.pdf [file pone.0299927.s008.pdf]

MAKERERE UNIVERSITY
COLLEGE OF HEALTH SCIENCES
SCHOOL OF MEDICINE
CHILD HEALTH AND DEVELOPMENT CENTRE

P. O. Box 6717 **Phone:** 256-414-541684, **Fax:** 256-414-531677 Kampala - Uganda
Email: chdc@chdc.mak.ac.ug **Website:** www.chdc.mak.ac.ug

Subject: Response to Reviewers - Manuscript PONE-D-23-16533R2

Dear Dr. Richard Ente Claros,

I trust this message finds you well. We appreciate the detailed feedback provided by the Editor and Reviewers on my manuscript titled "The impact of the Parenting for Respectability program on violent parenting and intimate partner relationships in Uganda: a pre-post study" (Manuscript ID: PONE-D-23-16533R2). We have found the reviewers comments very useful and have greatly helped to strengthen the manuscript.

We have prepared a Response to Reviewers, addressing each point raised by the Editor and Reviewer 3 as advised by the editor. Below, find responses the responses in a tabular format.

The revised Response to Reviewers letter is attached below for your perusal.

**Comment**	**Response**
Please revise your paper based on reviewer 3's comments.	This has been done as advised.
The article presents a comprehensive evaluation of the Parenting for Respectability Program (PfR), aiming to reduce violence against children and intimate partner violence in Uganda. The pre-post study design, while not without its limitations, provides initial evidence that PfR may be an effective intervention for improving parenting skills and reducing familial violence. The inclusion of both parents' and children's perspectives strengthens the findings and provides a multidimensional view of the program's impact.<br><br>A notable strength of the article is its detailed examination of various outcomes, including harsh parenting, dysfunctional partner relationships, and parenting self-efficacy. The large effect sizes reported, especially in	We thank the reviewer for this positive comment

reducing harsh parenting as reported by fathers, are significant. It suggests that the program is not only successful in engaging fathers but may also shift paternal behavior positively, which is often a challenging area in similar interventions.	
However, the study's design presents inherent limitations. The lack of a control group or randomized assignment to the intervention means that the observed changes cannot be conclusively attributed to PfR alone. There is also the possibility of self-report bias, particularly given the sensitive nature of the topics involved. The social desirability bias may have influenced participants to report outcomes that they perceive as more favorable or expected by the researchers.	We are glad to reviewer shares similar concerns with us about the design inherent limitations, and has provided insightful views. We have improved the description of the limitations and it reads as:<br><br>The study's main limitation is that the quasi-experimental pre-post evaluation design made it impossible to consider and control for the secular changes that might have occurred during the study period (40). Social desirability bias might have been exacerbated through the delivery of program messages over 16 sessions, leading participants to exaggerate their self-reported post-intervention data. There is also the possibility that given the sensitive nature of the topics involved, the participants may have tended to report outcomes that they perceive as more favorable or expected by the researchers. However, outcome data on parent-child and parent-parent relationships collected from children, who did not participate in the sessions, were likely to have greater validity since far less subject to social desirability bias. They too showed marked improvements in outcomes. Thus, the inclusion of both parents' and children's perspectives strengthens the findings and provides a multidimensional view of the program's impact.
The article's discussion on the larger effect of the program on older parents compared to younger ones is an interesting finding that could indicate the need for more targeted approaches in parenting programs. This could reflect differences in generational parenting styles or varying levels of receptivity to non-traditional parenting methods. Further research is needed to explore the reasons behind this age-related disparity in outcomes.	We agree with this suggestion for further research, and have noted it in the conclusion.<br><br>The results of this pre-post study suggest that the intervention warrants more rigorous evaluation through a randomized controlled trial. Further research is also needed to explore why the program had larger effects on older parents than younger ones, such as generational differences in parenting styles or receptivity to non-traditional parenting methods.
The medium effect sizes observed in reducing dysfunctional partner relationships and improving	Thank you for this comment from which we have drawn great insight on how to improve and expand

respectful child behavior are promising. These findings contribute to the growing body of evidence supporting the efficacy of family-based interventions in improving overall family dynamics and reducing potential for violence.<br><br>The article also notes the success of PfR in recruiting and retaining male participants, which is particularly commendable given the traditional challenges in male engagement in such programs. This success could serve as a model for similar interventions, although more details on the strategies employed to achieve high male participation would enhance the replicability of the program.	the original text. We have revised the section, providing additional description of the overall intervention strategy and approach, and it now reads as follows:<br><br>Our findings provide further evidence of the greater benefit of targeting both female and male parents. The rate of recruitment and retention of men in our study was considerably higher than reported in many trials, and offers great promise on the viability of programs to promote male engagement, and could serve as a model for similar interventions. Strategies employed to achieve high male participation included presenting the program as targeting fathers as much as it does mothers, and explaining the expected benefits of greater male involvement to fathers in their own right. Our program deliberately recruited parental couples, and this increased the number of fathers, and its delivery structure involving formation of separate groups for fathers and mothers, initially, allowed them to participate in both single sex in the first half of the program and then completing the program in mixed sex sessions. Single sex sessions offered safe space for both sexes to initially explore gender sensitive parenting concerns and spousal relationships without fear of being judged, and fathers greatly welcomed this model.<br><br>In general, intimate partner violence in Sub-Saharan Africa is perpetrated by men. The PfR approach recognizes this fact, but rather than focusing much attention on demonizing men as perpetrators of violence and thereby risk discouraging from participating in the programme, it appeals to men's positive motivation, and seeks to recruit them as allies in addressing family violence and poor parenting. The program's theoretical perspective is sensitive, recognizes men as interested parties, and promotes their core aspiration to achieve family respectability by encouraging positive practices around co-existence, shared family values, and father's roles, without reproducing existing predominant norms.
Moreover, the authors highlight the cultural sensitivity of	We find this comment great. We have provided

PfR and its alignment with national strategies, which likely contributes to its effectiveness. This underscores the importance of culturally grounded interventions in creating sustainable change within communities.<br><br>The discussion could be further enriched by addressing the potential for PfR to influence broader social and gender norms, given its impact on attitudes towards gender socialization. While the article acknowledges that changing these norms is a long-term endeavor, it would benefit from a deeper exploration of how the program's outcomes could contribute to this broader goal.	suggestions on how the PfR program influence gender and social norms at a broader level, with the following text:<br><br>The *PfR* intervention could influence broader social and gender norms at a macro level by: a) its community-based, inclusive approach; b) delivery as a universal prevention programme rather than targeted; c) addressing the long-term, intergenerational transfer of gender socialising norms; d) scaling up the programme nationally pending results from a cluster RCT; and e) combining the programme with other population level norm changing interventions.
In conclusion, while the study provides valuable insights into the effectiveness of PfR, it also opens up several avenues for further research. A future randomized controlled trial could confirm the causality of the observed outcomes, and a longitudinal study could assess the long-term sustainability of the program's impact. Additionally, expanding the discussion on how PfR could be adapted for different cultural contexts or age groups would be beneficial. Overall, the article makes a significant contribution to the literature on violence prevention and parenting programs in low-resource settings.	Thank you. This has been noted and addressed in the conclusion

Kind regards,

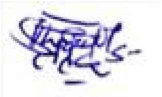

Dr. Godfrey Siu
