# Supplementary material for: The impact of the parenting for respectability programme on violent parenting and intimate partner relationships in Uganda: A pre-post study
Source: PLoS One. 2024 May 24;19(5):e0299927. doi: 10.1371/journal.pone.0299927 (PMC11125497; doi:10.1371/journal.pone.0299927)
Supplement: Attachment — Submitted filename: Response to Reviewers.docx [file pone.0299927.s006.docx]

PONE-D-23-16533

**The impact of the *Parenting for Respectability* program on violent parenting and intimate partner relationships in Uganda: a pre-post study**

| **Comment/Query** | **Response** |
| --- | --- |
| Thank you for submitting your manuscript to PLOS ONE. After careful consideration, we feel that it has merit but does not fully meet PLOS ONE’s publication criteria as it currently stands. Therefore, we invite you to submit a revised version of the manuscript that addresses the points raised during the review process. | Thank you for the review and consideration to publish this manuscript. We are glad to be invited to revise and resubmit the manuscript |
| Thank you for stating the following financial disclosure:  “No”  At this time, please address the following queries:  a) Please clarify the sources of funding (financial or material support) for your study. List the grants or organizations that supported your study, including funding received from your institution.  b) State what role the funders took in the study. If the funders had no role in your study, please state: “The funders had no role in study design, data collection and analysis, decision to publish, or preparation of the manuscript.”  c) If any authors received a salary from any of your funders, please state which authors and which funders.  d) If you did not receive any funding for this study, please state: “The authors received no specific funding for this work.”  Please include your amended statements within your cover letter; we will change the online submission form on your behalf. | The study received funding from the Sexual Violence Research Initiative (SVRI) and from the University of Glasgow Social and Public Health Sciences Unit. The funders did not have an active role in the study. However, the SVRI organized an international conference in which the preliminary study results were presented and critiqued by the participants, while the University of Glasgow funded a research methods course in the UK attended by the lead author. The authors’ salaries were paid by their respective institutions |
| In your Data Availability statement, you have not specified where the minimal data set underlying the results described in your manuscript can be found. PLOS defines a study's minimal data set as the underlying data used to reach the conclusions drawn in the manuscript and any additional data required to replicate the reported study findings in their entirety. All PLOS journals require that the minimal data set be made fully available. For more information about our data policy, please see <http://journals.plos.org/plosone/s/data-availability>.  Upon re-submitting your revised manuscript, please upload your study’s minimal underlying data set as either Supporting Information files or to a stable, public repository and include the relevant URLs, DOIs, or accession numbers within your revised cover letter. For a list of acceptable repositories, please see <http://journals.plos.org/plosone/s/data-availability#loc-recommended-repositories>. Any potentially identifying patient information must be fully anonymized.  Important: If there are ethical or legal restrictions to sharing your data publicly, please explain these restrictions in detail. Please see our guidelines for more information on what we consider unacceptable restrictions to publicly sharing data: <http://journals.plos.org/plosone/s/data-availability#loc-unacceptable-data-access-restrictions>. Note that it is not acceptable for the authors to be the sole named individuals responsible for ensuring data access. We will update your Data Availability statement to reflect the information you provide in your cover letter. | We appreciate the importance and potential value of making our data publicly available. However, we are currently unable to make these data publicly available for the following reasons (i) concerns around compromising participants’ privacy and breaching the consent agreement made with the participants. During data collection, participants signed consent forms and they were promised that their data would not be shared with anyone else other than the research team, and this was also the condition upon which the Institutional Review Board approved the study (ii) Our institutional data sharing policy places an embargo to publication of the dataset for 6 years, or until the main manuscripts have been published. However, we will gladly make the data available upon request through the Uganda Virus Research Institute’s IRB. The data maybe requested from Dr Tom Lutalo, Chair UVRI Research Ethics Committee via email: [directoruvri@uvri.go.ug](mailto:directoruvri@uvri.go.ug); phone: +256414320385/6 |
| Please amend your manuscript to include your abstract after the title page | This has been done |
| Please include a separate caption for each figure in your manuscript. | This has been done |
| Please include a separate caption for each figure in your manuscript. | This has been done |
| Please include your tables as part of your main manuscript and remove the individual files. Please note that supplementary tables (should remain/ be uploaded) as separate "supporting information" files | This has been done |
| Data analysis section. The data analysis does not contain enough details to let the reader fully understand the statistical models used. I am sure what the authors do is correct and has some merits, but the way they describe the statistical methodology is too concise. The statistical analysis section should be somehow extended. | We acknowledge that this section was indeed a bit too concise. We have now addressed this concern and expanded the analysis section. |
| Below are some specific examples of sentences that should be improved, even if the authors do not have to focus their attention on those sentences only. | The extended section addresses the specific issues as follows  a. As for the sentence:  We then conducted confirmatory factor analysis (CFA) and computed outcome scores with Cronbach’s Alphas [36]  The authors should add the variables (type of the variables) on which CFA is computed. They should also briefly say something about the motivation to compute both, CFA and Cronbach’s Alphas. In my knowledge, CFA is more related to validity whereas Cronbach’s Alpha is more related to reliability.  Frequencies for several socio-demographic and risk factors were calculated to assess their distribution. Based on results from the formative evaluation stage of the PfR, constructs were formed comprising of specific items. Data was collected on these constructs formed by the respective items.  We then conducted confirmatory factor analyses (CFA) to assess if the items within each construct in the current study still held together as in the formative evaluation stage. Each construct contained varied number of items ranging from 3 to 18 for both the primary and secondary study outcomes. and computed outcome scores with Cronbach’s Alphas (25). After the CFA we performed a reliability analysis using the Cronbach’s Alpha (25) method to ascertain if the items within a given construct conferred internal consistency. An alpha of at least 0.7 was indicative of good reliability.  Once the items within in each construct confirmed internal consistency, we generated scores for each construct/variables for both the primary and secondary outcomes calculated by summing the scores assigned by each respondent on all items per construct. The scores ranginged between 0 (e.g. never) to 4 (e.g. always).  We assessed if these scores were normally distributed because the methods we were planning to use for the follow-on analyses have the assumption of normality. We used exploratory analysis methods e.g. summary statistics (comparing the mean and median), histograms and symmetry plots to assess normality. The results confirmed that the scores passed the normality test |
| b. As for the sentence:  Full information maximum likelihood estimation method was used to account for missing data.  it is preliminary important to understand what kind of missing data the authors have. Depending on the type of missingness, the approach used may be different. | This has been addressed |
| Model validation. The authors use several statistical methods (confirmatory factor analysis, linear mixed models, and ANCOVA, just to cite the most relevant). All these methods are based on underlying assumptions. These assumptions should be first stated by the authors and then conveniently validated on the available data. Model validation (i.e., evaluating whether a chosen statistical model is appropriate or not) is just as important as model fitting in a good statistical analysis. | We appreciate and agree with this observation.  We have now improved this write-up with the following: All the models were validated to assess if they conferred goodness of fit to the data by computing fitted scores and comparing them with the scores for each construct. We also checked if the residuals were normally distributed. All analyses were run using the STATA software version 15 (StataCorp. 2017). |
| Further specific comments are directly annotated to the pdf of the paper in the attached file named “Specific Comments.pdf”. | We have seen this and are grateful to the reviewer for picking up these specific issues and editorials, which we have now addressed fully in the revised manuscript. |
|  |  |
