# Supplementary material for: The impact of the parenting for respectability programme on violent parenting and intimate partner relationships in Uganda: A pre-post study
Source: PLoS One. 2024 May 24;19(5):e0299927. doi: 10.1371/journal.pone.0299927 (PMC11125497; doi:10.1371/journal.pone.0299927)
Supplement: Attachment — Submitted filename: Reply_reviewer-PlosOne.docx [file pone.0299927.s007.docx]

The article presents a comprehensive evaluation of the Parenting for Respectability Program (PfR), aiming to reduce violence against children and intimate partner violence in Uganda. The pre-post study design, while not without its limitations, provides initial evidence that PfR may be an effective intervention for improving parenting skills and reducing familial violence. The inclusion of both parents' and children's perspectives strengthens the findings and provides a multidimensional view of the program's impact.

A notable strength of the article is its detailed examination of various outcomes, including harsh parenting, dysfunctional partner relationships, and parenting self-efficacy. The large effect sizes reported, especially in reducing harsh parenting as reported by fathers, are significant. It suggests that the program is not only successful in engaging fathers but may also shift paternal behavior positively, which is often a challenging area in similar interventions.

However, the study's design presents inherent limitations. The lack of a control group or randomized assignment to the intervention means that the observed changes cannot be conclusively attributed to PfR alone. There is also the possibility of self-report bias, particularly given the sensitive nature of the topics involved. The social desirability bias may have influenced participants to report outcomes that they perceive as more favorable or expected by the researchers.

The article's discussion on the larger effect of the program on older parents compared to younger ones is an interesting finding that could indicate the need for more targeted approaches in parenting programs. This could reflect differences in generational parenting styles or varying levels of receptivity to non-traditional parenting methods. Further research is needed to explore the reasons behind this age-related disparity in outcomes.

The medium effect sizes observed in reducing dysfunctional partner relationships and improving respectful child behavior are promising. These findings contribute to the growing body of evidence supporting the efficacy of family-based interventions in improving overall family dynamics and reducing potential for violence.

The article also notes the success of PfR in recruiting and retaining male participants, which is particularly commendable given the traditional challenges in male engagement in such programs. This success could serve as a model for similar interventions, although more details on the strategies employed to achieve high male participation would enhance the replicability of the program.

Moreover, the authors highlight the cultural sensitivity of PfR and its alignment with national strategies, which likely contributes to its effectiveness. This underscores the importance of culturally grounded interventions in creating sustainable change within communities.

The discussion could be further enriched by addressing the potential for PfR to influence broader social and gender norms, given its impact on attitudes towards gender socialization. While the article acknowledges that changing these norms is a long-term endeavor, it would benefit from a deeper exploration of how the program's outcomes could contribute to this broader goal.

In conclusion, while the study provides valuable insights into the effectiveness of PfR, it also opens up several avenues for further research. A future randomized controlled trial could confirm the causality of the observed outcomes, and a longitudinal study could assess the long-term sustainability of the program's impact. Additionally, expanding the discussion on how PfR could be adapted for different cultural contexts or age groups would be beneficial. Overall, the article makes a significant contribution to the literature on violence prevention and parenting programs in low-resource settings.
