# Supplementary material for: The impact of the parenting for respectability programme on violent parenting and intimate partner relationships in Uganda: A pre-post study
Source: PLoS One. 2024 May 24;19(5):e0299927. doi: 10.1371/journal.pone.0299927 (PMC11125497; doi:10.1371/journal.pone.0299927)
Supplement: Peer review history

# Peer review: Decision Letter 0

**DOI:** 10.1371/journal.pone.0299927.r001

**Authors:** Steve Zimmerman

27 Jul 2023

PONE-D-23-16533Impact of the Parenting for Respectability program on intimate partner violence and violence against children in Uganda: a pre-post studyPLOS ONE

Dear Dr. Siu,

Thank you for submitting your manuscript to PLOS ONE. After careful consideration, we feel that it has merit but does not fully meet PLOS ONE’s publication criteria as it currently stands. Therefore, we invite you to submit a revised version of the manuscript that addresses the points raised during the review process.

Please note that we have only been able to secure a single reviewer to assess your manuscript. We are issuing a decision on your manuscript at this point to prevent further delays in the evaluation of your manuscript. Please be aware that the editor who handles your revised manuscript might find it necessary to invite additional reviewers to assess this work once the revised manuscript is submitted. However, we will aim to proceed on the basis of this single review if possible. 

Please submit your revised manuscript by Sep 10 2023 11:59PM. If you will need more time than this to complete your revisions, please reply to this message or contact the journal office at plosone@plos.org. When you're ready to submit your revision, log on to https://www.editorialmanager.com/pone/ and select the 'Submissions Needing Revision' folder to locate your manuscript file.

Please include the following items when submitting your revised manuscript:

- A rebuttal letter that responds to each point raised by the academic editor and reviewer(s). You should upload this letter as a separate file labeled 'Response to Reviewers'.
- A marked-up copy of your manuscript that highlights changes made to the original version. You should upload this as a separate file labeled 'Revised Manuscript with Track Changes'.
- An unmarked version of your revised paper without tracked changes. You should upload this as a separate file labeled 'Manuscript'.

If you would like to make changes to your financial disclosure, please include your updated statement in your cover letter. Guidelines for resubmitting your figure files are available below the reviewer comments at the end of this letter.

If applicable, we recommend that you deposit your laboratory protocols in protocols.io to enhance the reproducibility of your results. Protocols.io assigns your protocol its own identifier (DOI) so that it can be cited independently in the future. For instructions see: https://journals.plos.org/plosone/s/submission-guidelines#loc-laboratory-protocols. Additionally, PLOS ONE offers an option for publishing peer-reviewed Lab Protocol articles, which describe protocols hosted on protocols.io. Read more information on sharing protocols at https://plos.org/protocols?utm_medium=editorial-email&utm_source=authorletters&utm_campaign=protocols.

We look forward to receiving your revised manuscript.

Kind regards,

Steve Zimmerman, PhD

Associate Editor, PLOS ONE

Journal requirements:

When submitting your revision, we need you to address these additional requirements.

1. Please ensure that your manuscript meets PLOS ONE's style requirements, including those for file naming. The PLOS ONE style templates can be found at

https://journals.plos.org/plosone/s/file?id=wjVg/PLOSOne_formatting_sample_main_body.pdf and

https://journals.plos.org/plosone/s/file?id=ba62/PLOSOne_formatting_sample_title_authors_affiliations.pdf

2. Thank you for stating the following financial disclosure:

“No”

At this time, please address the following queries:

a) Please clarify the sources of funding (financial or material support) for your study. List the grants or organizations that supported your study, including funding received from your institution.

b) State what role the funders took in the study. If the funders had no role in your study, please state: “The funders had no role in study design, data collection and analysis, decision to publish, or preparation of the manuscript.”

c) If any authors received a salary from any of your funders, please state which authors and which funders.

d) If you did not receive any funding for this study, please state: “The authors received no specific funding for this work.”

Please include your amended statements within your cover letter; we will change the online submission form on your behalf.

3. In your Data Availability statement, you have not specified where the minimal data set underlying the results described in your manuscript can be found. PLOS defines a study's minimal data set as the underlying data used to reach the conclusions drawn in the manuscript and any additional data required to replicate the reported study findings in their entirety. All PLOS journals require that the minimal data set be made fully available. For more information about our data policy, please see http://journals.plos.org/plosone/s/data-availability.

Upon re-submitting your revised manuscript, please upload your study’s minimal underlying data set as either Supporting Information files or to a stable, public repository and include the relevant URLs, DOIs, or accession numbers within your revised cover letter. For a list of acceptable repositories, please see http://journals.plos.org/plosone/s/data-availability#loc-recommended-repositories. Any potentially identifying patient information must be fully anonymized.

Important: If there are ethical or legal restrictions to sharing your data publicly, please explain these restrictions in detail. Please see our guidelines for more information on what we consider unacceptable restrictions to publicly sharing data: http://journals.plos.org/plosone/s/data-availability#loc-unacceptable-data-access-restrictions. Note that it is not acceptable for the authors to be the sole named individuals responsible for ensuring data access.

We will update your Data Availability statement to reflect the information you provide in your cover letter.

4. Please amend your manuscript to include your abstract after the title page.

5. Please include a separate caption for each figure in your manuscript.

6. Please include your tables as part of your main manuscript and remove the individual files. Please note that supplementary tables (should remain/ be uploaded) as separate "supporting information" files

[Note: HTML markup is below. Please do not edit.]

Reviewers' comments:

Reviewer's Responses to Questions

**Comments to the Author**

1. Is the manuscript technically sound, and do the data support the conclusions?

The manuscript must describe a technically sound piece of scientific research with data that supports the conclusions. Experiments must have been conducted rigorously, with appropriate controls, replication, and sample sizes. The conclusions must be drawn appropriately based on the data presented. 

Reviewer #1: Yes

**********

2. Has the statistical analysis been performed appropriately and rigorously? 

Reviewer #1: Yes

**********

3. Have the authors made all data underlying the findings in their manuscript fully available?

The PLOS Data policy requires authors to make all data underlying the findings described in their manuscript fully available without restriction, with rare exception (please refer to the Data Availability Statement in the manuscript PDF file). The data should be provided as part of the manuscript or its supporting information, or deposited to a public repository. For example, in addition to summary statistics, the data points behind means, medians and variance measures should be available. If there are restrictions on publicly sharing data—e.g. participant privacy or use of data from a third party—those must be specified.

Reviewer #1: Yes

**********

4. Is the manuscript presented in an intelligible fashion and written in standard English?

PLOS ONE does not copyedit accepted manuscripts, so the language in submitted articles must be clear, correct, and unambiguous. Any typographical or grammatical errors should be corrected at revision, so please note any specific errors here.

Reviewer #1: Yes

**********

5. Review Comments to the Author

Please use the space provided to explain your answers to the questions above. You may also include additional comments for the author, including concerns about dual publication, research ethics, or publication ethics. (Please upload your review as an attachment if it exceeds 20,000 characters)

Reviewer #1: The article is well-written. The statistical analysis of the paper is well-done enough. However, there are some aspects, those given below, the authors should deal with.

1. Data analysis section. The data analysis does not contain enough details to let the reader fully understand the statistical models used. I am sure what the authors do is correct and has some merits, but the way they describe the statistical methodology is too concise. The statistical analysis section should be somehow extended. Below are some specific examples of sentences that should be improved, even if the authors do not have to focus their attention on those sentences only.

a. As for the sentence:

We then conducted confirmatory factor analysis (CFA) and computed outcome scores with Cronbach’s Alphas [36]

The authors should add the variables (type of the variables) on which CFA is computed. They should also briefly say something about the motivation to compute both, CFA and Cronbach’s Alphas. In my knowledge, CFA is more related to validity whereas Cronbach’s Alpha is more related to reliability.

b. As for the sentence:

Full information maximum likelihood estimation method was used to account for missing data.

it is preliminary important to understand what kind of missing data the authors have. Depending on the type of missingness, the approach used may be different.

c. As for the sentence:

A total of 484 parents (n=269 mothers, 215 fathers) and 212 children (n=117 girls, 95 boys)

it is not clear to me the use of the symbol n. It seems that the authors use the same symbol for different stuff. They should be more precise about notation.

2. Model validation. The authors use several statistical methods (confirmatory factor analysis, linear mixed models, and ANCOVA, just to cite the most relevant). All these methods are based on underlying assumptions. These assumptions should be first stated by the authors and then conveniently validated on the available data. Model validation (i.e., evaluating whether a chosen statistical model is appropriate or not) is just as important as model fitting in a good statistical analysis.

3. Further specific comments are directly annotated to the pdf of the paper in the attached file named “Specific Comments.pdf”.

**********

6. PLOS authors have the option to publish the peer review history of their article (what does this mean?). If published, this will include your full peer review and any attached files.

If you choose “no”, your identity will remain anonymous but your review may still be made public.

**Do you want your identity to be public for this peer review?** For information about this choice, including consent withdrawal, please see our Privacy Policy.

Reviewer #1: No

**********

[NOTE: If reviewer comments were submitted as an attachment file, they will be attached to this email and accessible via the submission site. Please log into your account, locate the manuscript record, and check for the action link "View Attachments". If this link does not appear, there are no attachment files.]

While revising your submission, please upload your figure files to the Preflight Analysis and Conversion Engine (PACE) digital diagnostic tool, https://pacev2.apexcovantage.com/. PACE helps ensure that figures meet PLOS requirements. To use PACE, you must first register as a user. Registration is free. Then, login and navigate to the UPLOAD tab, where you will find detailed instructions on how to use the tool. If you encounter any issues or have any questions when using PACE, please email PLOS at figures@plos.org. Please note that Supporting Information files do not need this step.

Attachment Submitted filename: Specific Comments.pdf

# Author comment: Author response to Decision Letter 0

**DOI:** 10.1371/journal.pone.0299927.r002

4 Oct 2023

Thank you for submitting your manuscript to PLOS ONE. After careful consideration, we feel that it has merit but does not fully meet PLOS ONE’s publication criteria as it currently stands. Therefore, we invite you to submit a revised version of the manuscript that addresses the points raised during the review process. Thank you for the review and consideration to publish this manuscript. We are glad to be invited to revise and resubmit the manuscript.

Thank you for stating the following financial disclosure:

“No”

At this time, please address the following queries:

a) Please clarify the sources of funding (financial or material support) for your study. List the grants or organizations that supported your study, including funding received from your institution.

b) State what role the funders took in the study. If the funders had no role in your study, please state: “The funders had no role in study design, data collection and analysis, decision to publish, or preparation of the manuscript.”

c) If any authors received a salary from any of your funders, please state which authors and which funders.

d) If you did not receive any funding for this study, please state: “The authors received no specific funding for this work.”

Please include your amended statements within your cover letter; we will change the online submission form on your behalf.

 The study received funding from the Sexual Violence Research Initiative (SVRI) and from the University of Glasgow Social and Public Health Sciences Unit. The funders did not have an active role in the study. However, the SVRI organized an international conference in which the preliminary study results were presented and critiqued by the participants, while the University of Glasgow funded a research methods course in the UK attended by the lead author. The authors’ salaries were paid by their respective institutions. 

In your Data Availability statement, you have not specified where the minimal data set underlying the results described in your manuscript can be found. PLOS defines a study's minimal data set as the underlying data used to reach the conclusions drawn in the manuscript and any additional data required to replicate the reported study findings in their entirety. All PLOS journals require that the minimal data set be made fully available. For more information about our data policy, please see http://journals.plos.org/plosone/s/data-availability.

Upon re-submitting your revised manuscript, please upload your study’s minimal underlying data set as either Supporting Information files or to a stable, public repository and include the relevant URLs, DOIs, or accession numbers within your revised cover letter. For a list of acceptable repositories, please see http://journals.plos.org/plosone/s/data-availability#loc-recommended-repositories. Any potentially identifying patient information must be fully anonymized.

Important: If there are ethical or legal restrictions to sharing your data publicly, please explain these restrictions in detail. Please see our guidelines for more information on what we consider unacceptable restrictions to publicly sharing data: http://journals.plos.org/plosone/s/data-availability#loc-unacceptable-data-access-restrictions. Note that it is not acceptable for the authors to be the sole named individuals responsible for ensuring data access. We will update your Data Availability statement to reflect the information you provide in your cover letter.

 We appreciate the importance and potential value of making our data publicly available. However, we are currently unable to make these data publicly available for the following reasons (i) concerns around compromising participants’ privacy and breaching the consent agreement made with the participants. During data collection, participants signed consent forms and they were promised that their data would not be shared with anyone else other than the research team, and this was also the condition upon which the Institutional Review Board approved the study (ii) Our institutional data sharing policy places an embargo to publication of the dataset for 6 years, or until the main manuscripts have been published. However, we will gladly make the data available upon request through the School of Humanities and Social Sciences IRB, Makerere University 

Please amend your manuscript to include your abstract after the title page This has been done

Please include a separate caption for each figure in your manuscript.

 This has been done

Please include a separate caption for each figure in your manuscript.

 This has been done

Please include your tables as part of your main manuscript and remove the individual files. Please note that supplementary tables (should remain/ be uploaded) as separate "supporting information" files

 This has been done

Data analysis section. The data analysis does not contain enough details to let the reader fully understand the statistical models used. I am sure what the authors do is correct and has some merits, but the way they describe the statistical methodology is too concise. The statistical analysis section should be somehow extended. 

 We acknowledge that this section was indeed a bit too concise. We have now addressed this concern and expanded the analysis section. 

Below are some specific examples of sentences that should be improved, even if the authors do not have to focus their attention on those sentences only.

 The extended section addresses the specific issues as follows 

a. As for the sentence:

We then conducted confirmatory factor analysis (CFA) and computed outcome scores with Cronbach’s Alphas [36]

The authors should add the variables (type of the variables) on which CFA is computed. They should also briefly say something about the motivation to compute both, CFA and Cronbach’s Alphas. In my knowledge, CFA is more related to validity whereas Cronbach’s Alpha is more related to reliability.

Frequencies for several socio-demographic and risk factors were calculated to assess their distribution. Based on results from the formative evaluation stage of the PfR, constructs were formed comprising of specific items. Data was collected on these constructs formed by the respective items. 

We then conducted confirmatory factor analyses (CFA) to assess if the items within each construct in the current study still held together as in the formative evaluation stage. Each construct contained varied number of items ranging from 3 to 18 for both the primary and secondary study outcomes. and computed outcome scores with Cronbach’s Alphas (25). After the CFA we performed a reliability analysis using the Cronbach’s Alpha (25) method to ascertain if the items within a given construct conferred internal consistency. An alpha of at least 0.7 was indicative of good reliability.

Once the items within in each construct confirmed internal consistency, we generated scores for each construct/variables for both the primary and secondary outcomes calculated by summing the scores assigned by each respondent on all items per construct. The scores ranginged between 0 (e.g. never) to 4 (e.g. always).

We assessed if these scores were normally distributed because the methods we were planning to use for the follow-on analyses have the assumption of normality. We used exploratory analysis methods e.g. summary statistics (comparing the mean and median), histograms and symmetry plots to assess normality. The results confirmed that the scores passed the normality test.

b. As for the sentence:

Full information maximum likelihood estimation method was used to account for missing data.

it is preliminary important to understand what kind of missing data the authors have. Depending on the type of missingness, the approach used may be different.

 This has been addressed

Model validation. The authors use several statistical methods (confirmatory factor analysis, linear mixed models, and ANCOVA, just to cite the most relevant). All these methods are based on underlying assumptions. These assumptions should be first stated by the authors and then conveniently validated on the available data. Model validation (i.e., evaluating whether a chosen statistical model is appropriate or not) is just as important as model fitting in a good statistical analysis.

 We appreciate and agree with this observation. 

We have now improved this write-up with the following: All the models were validated to assess if they conferred goodness of fit to the data by computing fitted scores and comparing them with the scores for each construct. We also checked if the residuals were normally distributed. All analyses were run using the STATA software version 15 (StataCorp. 2017).

Further specific comments are directly annotated to the pdf of the paper in the attached file named “Specific Comments.pdf”.

 We have seen this and are grateful to the reviewer for picking up these specific issues and editorials, which we have now addressed fully in the revised manuscript.

Attachment Submitted filename: Response to Reviewers.docx

# Peer review: Decision Letter 1

**DOI:** 10.1371/journal.pone.0299927.r003

**Authors:** Mu-Hong Chen

20 Nov 2023

PONE-D-23-16533R1The impact of the Parenting for Respectability program on violent parenting and intimate partner relationships in Uganda: a pre-post studyPLOS ONE

Dear Dr. Siu,

Thank you for submitting your manuscript to PLOS ONE. After careful consideration, we feel that it has merit but does not fully meet PLOS ONE’s publication criteria as it currently stands. Therefore, we invite you to submit a revised version of the manuscript that addresses the points raised during the review process.

Please submit your revised manuscript by Jan 04 2024 11:59PM. If you will need more time than this to complete your revisions, please reply to this message or contact the journal office at plosone@plos.org. When you're ready to submit your revision, log on to https://www.editorialmanager.com/pone/ and select the 'Submissions Needing Revision' folder to locate your manuscript file.

Please include the following items when submitting your revised manuscript:

- A rebuttal letter that responds to each point raised by the academic editor and reviewer(s). You should upload this letter as a separate file labeled 'Response to Reviewers'.
- A marked-up copy of your manuscript that highlights changes made to the original version. You should upload this as a separate file labeled 'Revised Manuscript with Track Changes'.
- An unmarked version of your revised paper without tracked changes. You should upload this as a separate file labeled 'Manuscript'.

If you would like to make changes to your financial disclosure, please include your updated statement in your cover letter. Guidelines for resubmitting your figure files are available below the reviewer comments at the end of this letter.

If applicable, we recommend that you deposit your laboratory protocols in protocols.io to enhance the reproducibility of your results. Protocols.io assigns your protocol its own identifier (DOI) so that it can be cited independently in the future. For instructions see: https://journals.plos.org/plosone/s/submission-guidelines#loc-laboratory-protocols. Additionally, PLOS ONE offers an option for publishing peer-reviewed Lab Protocol articles, which describe protocols hosted on protocols.io. Read more information on sharing protocols at https://plos.org/protocols?utm_medium=editorial-email&utm_source=authorletters&utm_campaign=protocols.

We look forward to receiving your revised manuscript.

Kind regards,

Mu-Hong Chen, M.D., Ph.D.

Academic Editor

PLOS ONE

Additional Editor Comments:

Please revise your paper based on reviewer 3's comments.

The article presents a comprehensive evaluation of the Parenting for Respectability Program (PfR), aiming to reduce violence against children and intimate partner violence in Uganda. The pre-post study design, while not without its limitations, provides initial evidence that PfR may be an effective intervention for improving parenting skills and reducing familial violence. The inclusion of both parents' and children's perspectives strengthens the findings and provides a multidimensional view of the program's impact.

A notable strength of the article is its detailed examination of various outcomes, including harsh parenting, dysfunctional partner relationships, and parenting self-efficacy. The large effect sizes reported, especially in reducing harsh parenting as reported by fathers, are significant. It suggests that the program is not only successful in engaging fathers but may also shift paternal behavior positively, which is often a challenging area in similar interventions.

However, the study's design presents inherent limitations. The lack of a control group or randomized assignment to the intervention means that the observed changes cannot be conclusively attributed to PfR alone. There is also the possibility of self-report bias, particularly given the sensitive nature of the topics involved. The social desirability bias may have influenced participants to report outcomes that they perceive as more favorable or expected by the researchers.

The article's discussion on the larger effect of the program on older parents compared to younger ones is an interesting finding that could indicate the need for more targeted approaches in parenting programs. This could reflect differences in generational parenting styles or varying levels of receptivity to non-traditional parenting methods. Further research is needed to explore the reasons behind this age-related disparity in outcomes.

The medium effect sizes observed in reducing dysfunctional partner relationships and improving respectful child behavior are promising. These findings contribute to the growing body of evidence supporting the efficacy of family-based interventions in improving overall family dynamics and reducing potential for violence.

The article also notes the success of PfR in recruiting and retaining male participants, which is particularly commendable given the traditional challenges in male engagement in such programs. This success could serve as a model for similar interventions, although more details on the strategies employed to achieve high male participation would enhance the replicability of the program.

Moreover, the authors highlight the cultural sensitivity of PfR and its alignment with national strategies, which likely contributes to its effectiveness. This underscores the importance of culturally grounded interventions in creating sustainable change within communities.

The discussion could be further enriched by addressing the potential for PfR to influence broader social and gender norms, given its impact on attitudes towards gender socialization. While the article acknowledges that changing these norms is a long-term endeavor, it would benefit from a deeper exploration of how the program's outcomes could contribute to this broader goal.

In conclusion, while the study provides valuable insights into the effectiveness of PfR, it also opens up several avenues for further research. A future randomized controlled trial could confirm the causality of the observed outcomes, and a longitudinal study could assess the long-term sustainability of the program's impact. Additionally, expanding the discussion on how PfR could be adapted for different cultural contexts or age groups would be beneficial. Overall, the article makes a significant contribution to the literature on violence prevention and parenting programs in low-resource settings.

[Note: HTML markup is below. Please do not edit.]

Reviewers' comments:

Reviewer's Responses to Questions

**Comments to the Author**

1. If the authors have adequately addressed your comments raised in a previous round of review and you feel that this manuscript is now acceptable for publication, you may indicate that here to bypass the “Comments to the Author” section, enter your conflict of interest statement in the “Confidential to Editor” section, and submit your "Accept" recommendation.

Reviewer #1: All comments have been addressed

Reviewer #2: All comments have been addressed

Reviewer #3: All comments have been addressed

**********

2. Is the manuscript technically sound, and do the data support the conclusions?

The manuscript must describe a technically sound piece of scientific research with data that supports the conclusions. Experiments must have been conducted rigorously, with appropriate controls, replication, and sample sizes. The conclusions must be drawn appropriately based on the data presented. 

Reviewer #1: Yes

Reviewer #2: Yes

Reviewer #3: Yes

**********

3. Has the statistical analysis been performed appropriately and rigorously? 

Reviewer #1: Yes

Reviewer #2: Yes

Reviewer #3: Yes

**********

4. Have the authors made all data underlying the findings in their manuscript fully available?

The PLOS Data policy requires authors to make all data underlying the findings described in their manuscript fully available without restriction, with rare exception (please refer to the Data Availability Statement in the manuscript PDF file). The data should be provided as part of the manuscript or its supporting information, or deposited to a public repository. For example, in addition to summary statistics, the data points behind means, medians and variance measures should be available. If there are restrictions on publicly sharing data—e.g. participant privacy or use of data from a third party—those must be specified.

Reviewer #1: Yes

Reviewer #2: No

Reviewer #3: Yes

**********

5. Is the manuscript presented in an intelligible fashion and written in standard English?

PLOS ONE does not copyedit accepted manuscripts, so the language in submitted articles must be clear, correct, and unambiguous. Any typographical or grammatical errors should be corrected at revision, so please note any specific errors here.

Reviewer #1: Yes

Reviewer #2: Yes

Reviewer #3: Yes

**********

6. Review Comments to the Author

Please use the space provided to explain your answers to the questions above. You may also include additional comments for the author, including concerns about dual publication, research ethics, or publication ethics. (Please upload your review as an attachment if it exceeds 20,000 characters)

Reviewer #1: (No Response)

Reviewer #2: It is a relevant study because it contributes to understanding the importance of intervention programs in parental practices in a context of violence. The authors made the modifications suggested previously.

ABSTRACT

It is well structured and written in a concise and easy-to-read form, highlighting the main points of the article.

INTRODUCTION

It presents the study problem in a consistent and well-structured manner, placing the significance of the study.

METHODS

They were described in a detailed and clear way.

Os autores explicam porque não podem disponibilizar publicamente todos os dados

RESULTS

The results are described and presented appropriately in the tables and figures.

DISCUSSION AND CONCLUSIN

The discussion and conclusion are consistent with the results found.

REFERENCES

They are organized properly, but it is necessary to check, because some references are incomplete (example: ref nº 14).

Reviewer #3: The article presents a comprehensive evaluation of the Parenting for Respectability Program (PfR), aiming to reduce violence against children and intimate partner violence in Uganda. The pre-post study design, while not without its limitations, provides initial evidence that PfR may be an effective intervention for improving parenting skills and reducing familial violence. The inclusion of both parents' and children's perspectives strengthens the findings and provides a multidimensional view of the program's impact.

A notable strength of the article is its detailed examination of various outcomes, including harsh parenting, dysfunctional partner relationships, and parenting self-efficacy. The large effect sizes reported, especially in reducing harsh parenting as reported by fathers, are significant. It suggests that the program is not only successful in engaging fathers but may also shift paternal behavior positively, which is often a challenging area in similar interventions.

However, the study's design presents inherent limitations. The lack of a control group or randomized assignment to the intervention means that the observed changes cannot be conclusively attributed to PfR alone. There is also the possibility of self-report bias, particularly given the sensitive nature of the topics involved. The social desirability bias may have influenced participants to report outcomes that they perceive as more favorable or expected by the researchers.

The article's discussion on the larger effect of the program on older parents compared to younger ones is an interesting finding that could indicate the need for more targeted approaches in parenting programs. This could reflect differences in generational parenting styles or varying levels of receptivity to non-traditional parenting methods. Further research is needed to explore the reasons behind this age-related disparity in outcomes.

The medium effect sizes observed in reducing dysfunctional partner relationships and improving respectful child behavior are promising. These findings contribute to the growing body of evidence supporting the efficacy of family-based interventions in improving overall family dynamics and reducing potential for violence.

The article also notes the success of PfR in recruiting and retaining male participants, which is particularly commendable given the traditional challenges in male engagement in such programs. This success could serve as a model for similar interventions, although more details on the strategies employed to achieve high male participation would enhance the replicability of the program.

Moreover, the authors highlight the cultural sensitivity of PfR and its alignment with national strategies, which likely contributes to its effectiveness. This underscores the importance of culturally grounded interventions in creating sustainable change within communities.

The discussion could be further enriched by addressing the potential for PfR to influence broader social and gender norms, given its impact on attitudes towards gender socialization. While the article acknowledges that changing these norms is a long-term endeavor, it would benefit from a deeper exploration of how the program's outcomes could contribute to this broader goal.

In conclusion, while the study provides valuable insights into the effectiveness of PfR, it also opens up several avenues for further research. A future randomized controlled trial could confirm the causality of the observed outcomes, and a longitudinal study could assess the long-term sustainability of the program's impact. Additionally, expanding the discussion on how PfR could be adapted for different cultural contexts or age groups would be beneficial. Overall, the article makes a significant contribution to the literature on violence prevention and parenting programs in low-resource settings.

**********

7. PLOS authors have the option to publish the peer review history of their article (what does this mean?). If published, this will include your full peer review and any attached files.

If you choose “no”, your identity will remain anonymous but your review may still be made public.

**Do you want your identity to be public for this peer review?** For information about this choice, including consent withdrawal, please see our Privacy Policy.

Reviewer #1: No

Reviewer #2: **Yes: **Elisabete Pereira Silva

Reviewer #3: No

**********

[NOTE: If reviewer comments were submitted as an attachment file, they will be attached to this email and accessible via the submission site. Please log into your account, locate the manuscript record, and check for the action link "View Attachments". If this link does not appear, there are no attachment files.]

While revising your submission, please upload your figure files to the Preflight Analysis and Conversion Engine (PACE) digital diagnostic tool, https://pacev2.apexcovantage.com/. PACE helps ensure that figures meet PLOS requirements. To use PACE, you must first register as a user. Registration is free. Then, login and navigate to the UPLOAD tab, where you will find detailed instructions on how to use the tool. If you encounter any issues or have any questions when using PACE, please email PLOS at figures@plos.org. Please note that Supporting Information files do not need this step.

Attachment Submitted filename: Reply_reviewer-PlosOne.docx

# Author comment: Author response to Decision Letter 1

**DOI:** 10.1371/journal.pone.0299927.r004

11 Jan 2024

Comment Response

Please revise your paper based on reviewer 3's comments.

 This has been done as advised.

The article presents a comprehensive evaluation of the Parenting for Respectability Program (PfR), aiming to reduce violence against children and intimate partner violence in Uganda. The pre-post study design, while not without its limitations, provides initial evidence that PfR may be an effective intervention for improving parenting skills and reducing familial violence. The inclusion of both parents' and children's perspectives strengthens the findings and provides a multidimensional view of the program's impact.

A notable strength of the article is its detailed examination of various outcomes, including harsh parenting, dysfunctional partner relationships, and parenting self-efficacy. The large effect sizes reported, especially in reducing harsh parenting as reported by fathers, are significant. It suggests that the program is not only successful in engaging fathers but may also shift paternal behavior positively, which is often a challenging area in similar interventions.

 We thank the reviewer for this positive comment

However, the study's design presents inherent limitations. The lack of a control group or randomized assignment to the intervention means that the observed changes cannot be conclusively attributed to PfR alone. There is also the possibility of self-report bias, particularly given the sensitive nature of the topics involved. The social desirability bias may have influenced participants to report outcomes that they perceive as more favorable or expected by the researchers.

 We are glad to reviewer shares similar concerns with us about the design inherent limitations, and has provided insightful views. We have improved the description of the limitations and it reads as: 

The study’s main limitation is that the quasi-experimental pre-post evaluation design made it impossible to consider and control for the secular changes that might have occurred during the study period (40). Social desirability bias might have been exacerbated through the delivery of program messages over 16 sessions, leading participants to exaggerate their self-reported post-intervention data. There is also the possibility that given the sensitive nature of the topics involved, the participants may have tended to report outcomes that they perceive as more favorable or expected by the researchers. However, outcome data on parent-child and parent-parent relationships collected from children, who did not participate in the sessions, were likely to have greater validity since far less subject to social desirability bias. They too showed marked improvements in outcomes. Thus, the inclusion of both parents' and children's perspectives strengthens the findings and provides a multidimensional view of the program's impact.

The article's discussion on the larger effect of the program on older parents compared to younger ones is an interesting finding that could indicate the need for more targeted approaches in parenting programs. This could reflect differences in generational parenting styles or varying levels of receptivity to non-traditional parenting methods. Further research is needed to explore the reasons behind this age-related disparity in outcomes.

 We agree with this suggestion for further research, and have noted it in the conclusion. 

The results of this pre-post study suggest that the intervention warrants more rigorous evaluation through a randomized controlled trial. Further research is also needed to explore why the program had larger effects on older parents than younger ones, such as generational differences in parenting styles or receptivity to non-traditional parenting methods.

The medium effect sizes observed in reducing dysfunctional partner relationships and improving respectful child behavior are promising. These findings contribute to the growing body of evidence supporting the efficacy of family-based interventions in improving overall family dynamics and reducing potential for violence.

The article also notes the success of PfR in recruiting and retaining male participants, which is particularly commendable given the traditional challenges in male engagement in such programs. This success could serve as a model for similar interventions, although more details on the strategies employed to achieve high male participation would enhance the replicability of the program.

 Thank you for this comment from which we have drawn great insight on how to improve and expand the original text. We have revised the section, providing additional description of the overall intervention strategy and approach, and it now reads as follows: 

Our findings provide further evidence of the greater benefit of targeting both female and male parents. The rate of recruitment and retention of men in our study was considerably higher than reported in many trials, and offers great promise on the viability of programs to promote male engagement, and could serve as a model for similar interventions. Strategies employed to achieve high male participation included presenting the program as targeting fathers as much as it does mothers, and explaining the expected benefits of greater male involvement to fathers in their own right. Our program deliberately recruited parental couples, and this increased the number of fathers, and its delivery structure involving formation of separate groups for fathers and mothers, initially, allowed them to participate in both single sex in the first half of the program and then completing the program in mixed sex sessions. Single sex sessions offered safe space for both sexes to initially explore gender sensitive parenting concerns and spousal relationships without fear of being judged, and fathers greatly welcomed this model. 

In general, intimate partner violence in Sub-Saharan Africa is perpetrated by men. The PfR approach recognizes this fact, but rather than focusing much attention on demonizing men as perpetrators of violence and thereby risk discouraging from participating in the programme, it appeals to men’s positive motivation, and seeks to recruit them as allies in addressing family violence and poor parenting. The program’s theoretical perspective is sensitive, recognizes men as interested parties, and promotes their core aspiration to achieve family respectability by encouraging positive practices around co-existence, shared family values, and father’s roles, without reproducing existing predominant norms.

Moreover, the authors highlight the cultural sensitivity of PfR and its alignment with national strategies, which likely contributes to its effectiveness. This underscores the importance of culturally grounded interventions in creating sustainable change within communities.

The discussion could be further enriched by addressing the potential for PfR to influence broader social and gender norms, given its impact on attitudes towards gender socialization. While the article acknowledges that changing these norms is a long-term endeavor, it would benefit from a deeper exploration of how the program's outcomes could contribute to this broader goal.

 We find this comment great. We have provided suggestions on how the PfR program influence gender and social norms at a broader level, with the following text: 

The PfR intervention could influence broader social and gender norms at a macro level by: a) its community-based, inclusive approach; b) delivery as a universal prevention programme rather than targeted; c) addressing the long-term, intergenerational transfer of gender socialising norms; d) scaling up the programme nationally pending results from a cluster RCT; and e) combining the programme with other population level norm changing interventions. 

In conclusion, while the study provides valuable insights into the effectiveness of PfR, it also opens up several avenues for further research. A future randomized controlled trial could confirm the causality of the observed outcomes, and a longitudinal study could assess the long-term sustainability of the program's impact. Additionally, expanding the discussion on how PfR could be adapted for different cultural contexts or age groups would be beneficial. Overall, the article makes a significant contribution to the literature on violence prevention and parenting programs in low-resource settings.

 Thank you. We plan to undertake the conclusive recommendations made as soon as possible.

Attachment Submitted filename: Response to Reviewers letter.pdf

# Editor report: Decision Letter 2

**DOI:** 10.1371/journal.pone.0299927.r005

**Authors:** Mu-Hong Chen

19 Feb 2024

The impact of the Parenting for Respectability program on violent parenting and intimate partner relationships in Uganda: a pre-post study

PONE-D-23-16533R2

Dear Dr. Godfrey Siu,

We’re pleased to inform you that your manuscript has been judged scientifically suitable for publication and will be formally accepted for publication once it meets all outstanding technical requirements.

Within one week, you’ll receive an e-mail detailing the required amendments. When these have been addressed, you’ll receive a formal acceptance letter and your manuscript will be scheduled for publication.

An invoice for payment will follow shortly after the formal acceptance. To ensure an efficient process, please log into Editorial Manager at http://www.editorialmanager.com/pone/, click the 'Update My Information' link at the top of the page, and double check that your user information is up-to-date. If you have any billing related questions, please contact our Author Billing department directly at authorbilling@plos.org.

If your institution or institutions have a press office, please notify them about your upcoming paper to help maximize its impact. If they’ll be preparing press materials, please inform our press team as soon as possible -- no later than 48 hours after receiving the formal acceptance. Your manuscript will remain under strict press embargo until 2 pm Eastern Time on the date of publication. For more information, please contact onepress@plos.org.

Kind regards,

Mu-Hong Chen, M.D., Ph.D.

Academic Editor

PLOS ONE

Additional Editor Comments (optional):

Reviewers' comments:

# Editor report: Acceptance letter

**DOI:** 10.1371/journal.pone.0299927.r006

**Authors:** Mu-Hong Chen

5 Apr 2024

PONE-D-23-16533R2 

PLOS ONE

Dear Dr. Siu, 

I'm pleased to inform you that your manuscript has been deemed suitable for publication in PLOS ONE. Congratulations! Your manuscript is now being handed over to our production team.

At this stage, our production department will prepare your paper for publication. This includes ensuring the following:

* All references, tables, and figures are properly cited

* All relevant supporting information is included in the manuscript submission,

* There are no issues that prevent the paper from being properly typeset

If revisions are needed, the production department will contact you directly to resolve them. If no revisions are needed, you will receive an email when the publication date has been set. At this time, we do not offer pre-publication proofs to authors during production of the accepted work. Please keep in mind that we are working through a large volume of accepted articles, so please give us a few weeks to review your paper and let you know the next and final steps. 

Lastly, if your institution or institutions have a press office, please let them know about your upcoming paper now to help maximize its impact. If they'll be preparing press materials, please inform our press team within the next 48 hours. Your manuscript will remain under strict press embargo until 2 pm Eastern Time on the date of publication. For more information, please contact onepress@plos.org.

If we can help with anything else, please email us at customercare@plos.org.

Thank you for submitting your work to PLOS ONE and supporting open access. 

Kind regards, 

PLOS ONE Editorial Office Staff

on behalf of

Dr. Mu-Hong Chen 

Academic Editor

PLOS ONE
